# MXD3 as an Immunological and Prognostic Factor From Pancancer Analysis

**DOI:** 10.3389/fmolb.2021.702206

**Published:** 2021-11-11

**Authors:** Xiaoyu Zhang, Xiaoqin He, Yue Li, Yangtao Xu, Wenliang Chen, Xin Liu, Xinyao Hu, Lin Xiong, Ximing Xu

**Affiliations:** ^1^ Cancer Center, Renmin Hospital of Wuhan University, Wuhan, China; ^2^ Department of Pathology, Renmin Hospital of Wuhan University, Wuhan, China

**Keywords:** MXD3, cancer, prognosis, methylation, immune, immune infiltrating cell

## Abstract

MAX dimerization protein 3 (MXD3), a transcriptional regulator of the MXD3 superfamily, is a part of the MYC–MAX–MXD network. However, its role in tumors has been reported in several cancers, such as B-cell acute lymphoblastic leukemia, medulloblastoma, neuroblastoma, and glioblastoma. Based on TCGA and GEO data, our first pancancer study of MXD3 confirmed the high expression of MXD3 in cancer tissues. Our results revealed that patients suffering from cancers with higher MXD3 expression had poor OS, DSS, DFI, and PFI. We further explored the methylation status of the MXD3 gene body and gene promoter in cancer. Patients with a higher MXD3 gene body have better OS, while the prognosis of patients with a high MXD3 promoter is more complex. We also verified the differential expression of three clinical phenotypes of MXD3: age, sex, and tumor stage, in a variety of tumors, suggesting a correlation between MXD3 and clinical characteristics. We explored the negative relationship between MXD3 and TMB and MSI in most types of cancer, indicating the poor prognosis of patients with high MXD3 expression. We further investigated the relationship between MXD3 and immune infiltrating cells and identified the relationship between MXD3 and immune genes, immunosuppressive genes, and antigen-presenting genes. All of the above findings established a solid relationship between MXD3 and the immune environment and immune cells. These results demonstrated that MXD3 might also be a potential immune factor. We also found a higher expression of MXD3 and promoter according to the increasing glioma WHO grade or histologic types. Glioma patients with high MXD3 or MXD3 promoter expression had poor survival. Finally, we used IHC to verify the higher expression of MXD3 in glioma samples compared to normal samples. Our study shows that MXD3, as a poor prognostic factor, plays a significant role in many cancers, especially glioma. Although more clinical evidence for MXD3 as a clinical therapeutic target and an immunotherapy site is needed, MXD3 can play an important guiding role in multiple clinical treatments, including immunotherapy and demethylation therapy.

## Background

As a major mortality disease of humans, cancer threatens human health and affects the quality of life of patients. Additionally, cancer places a heavy economic burden on patients (Bray et al., 2018). Due to the recent increase in immunotherapy, researchers have reconsidered the role of immunity in cancer. Immune infiltrates are related to genetic alterations, clinical features, and viral infection status of patients (Pleasance et al., 2010a; Li et al., 2016). Genetic changes induce carcinogenesis, and large-scale parallel sequencing made it possible to systematically record this variation in the whole genome (Pleasance et al., 2010a; Pleasance et al., 2010b). Hence, for the development of cancer patient datasets, such as TCGA, it is easy to analyze the correlation between gene differences and clinical features and immune status.

MXD3 (MAX dimerization protein 3), a part of the MYC–MAX–MXD network, encodes a member of the Myc superfamily of basic helix–loop–helix leucine zipper transcriptional regulators. The network significantly affects the proliferation, differentiation, and apoptosis of organisms (Grandori et al., 2000). By forming the two heterocomplexes MYC–MAX and MXD–MAX, the network creates opposing effects on the combination of DNA and the E-box promoter sequences (Ayer et al., 1993). In general, recruitment of transcriptional cofactors by the MYC–MAX complex favors transcription of genes that promote proliferation (Kretzner et al., 1992). However, the MXD–MAX complex recruits transcriptional repressive factors to depress genes to promote differentiation (Ayer et al., 1996).

MXD3 has been reported as a vital factor in neuroblastoma (Barisone et al., 2012). In both mouse models and patients with medulloblastoma, MXD3 is upregulated. Knockdown of MXD3 caused a decrease in cell number *in vitro* (Ngo et al., 2014). MXD3 protein knockdown induced by MXD3 siRNA nanocomplexes results in apoptosis (Duong et al., 2017). These results suggest that MXD3 is an anti-apoptotic factor (Yoshida et al., 2020). Furthermore, the anti-apoptotic function of MXD3 has been identified in glioblastoma (Ngo et al., 2019) and B-cell acute lymphoblastic leukemia (Barisone et al., 2015). As a targeted therapeutic site, MXD3 enhances the killing of cancer cells and reduces the toxicity suffered by normal cells (Satake et al., 2014).

These results indicated the potential of MXD3 as a therapeutic target (Satake et al., 2014; Duong et al., 2017). Combining patient data from databases such as TCGA and GEO may elucidate the cancer landscape of MXD3 expression and eventually contribute to the precision treatment of cancer patients.

## Methods

### Data Processing

From UCSC Xena (https://xena.ucsc.edu/), we downloaded clinical phenotype data for 33 tumors, along with gene expression data, tumor mutation data, and microsatellite instability data. We used R software (Version 4.0.3; https://www.R-project.org) and Strawberry Perl (5.30.0.1) to process the data.

### MXD3 Expression

The exon and normal tissues of MXD3 expression data were obtained from the GTEx portal (https://www.gtexportal.org). The types of cancer with MXD3 expression data were obtained from Oncomine (https://www.oncomine.org). We obtained the mutation status from cBioPortal (http://www.cbioportal.org/). We processed TCGA MXD3 expression data by Perl and the R “limma” package and used the “ggplot2” package to draw boxplots.

### Analysis of the Relationships Between MXD3, Prognosis, and Clinical Phenotype

Overall survival (OS), disease-specific survival (DSS), disease-free interval (DFI), and progression-free interval (PFI) are classic clinical indicators that were analyzed with R. We used the Kaplan–Meier method and log-rank test for survival analyses of each cancer type. Moreover, we used the R packages “survival” and “forestplot” to uncover the indicators mentioned above. We drew survival curves with the following two R packages: “survival” and “survminer.”

We explored the relationship between the three clinical phenotypes and MXD3 mRNA expression data concerning tumor stage, age, and sex. We divided the age group of patients into two subgroups: 65 years old and above and below 65 years. The “limma” and “ggplot2” packages were used to determine the correlation.

### Analysis of MXD3 Expression With OS From GEO

We obtained GEO datasets from the Biomedical Informatics Institute (BII) (http://bioinfo.henu.edu.cn/Index.html) and calculated the OS of 26 types of cancer in the two groups, divided by the median MXD3 expression. We obtained the OS of GEO datasets by PrognoScan (http://gibk21.bse.kyutech.ac.jp/PrognoScan/index.html). And we showed the results of survival analysis for those cancers with positive results where the sample size was greater than 50.

### Analysis of MXD3 Expression With OS in ICI Cohort

We used the CAMOIP web tool (https://www.camoip.net/) to reveal the relationship between MXD3 expression in immune checkpoint inhibitor treatment and patient prognosis. The data of CAMOIP were coming from the GEO datasets.

### Correlation of MXD3 Expression With DNA Methylation

DNA methylation is a form of DNA chemical modification, and as an essential regulator of gene transcription, it can be carcinogenic. We used the web tool MethSurv, of which the data were derived from TCGA, to analyze MXD3 methylation in OS.

### Correlation of MXD3 Expression With Tumor Mutation Burden, Tumor Microsatellite Instability, and Mismatch Repair Gene Expression

We downloaded TMB and MSI data as mentioned above. We used the Pearson method to quantify the relationship between MXD3 expression and TMB and MSI for each cancer. We displayed the results in the radar plot using the “fbsm” package. We downloaded the mismatch repair gene list from the Gene Set Enrichment Analysis (GSEA) website. We analyzed the coexpression between this gene set and MXD3 by R and presented the results as a heatmap *via* the “ggplot2” package.

### Relationship Between MXD3 Expression and Immunity

Estimation of stromal and immune cells in malignant tumor tissues using expression data (ESTIMATE) is a method for inferring the degree of infiltration of stromal or immune cells into tumors using existing gene expression profiles. We assessed the overall immune landscape of different cancers using the “estimate” package. We presented the results as boxplots. CIBERSORT is a tool that estimates immune scores of immune cells based on gene expression status. We used this tool to capture the distribution of immune cells across cancers. We then used plots to show the correlation of MXD3 with infiltrating cells. All of the above was performed with the “cibersort” and “ggplot” packages.

### Coexpression of MXD3 With Immune Pathway–Related Genes

We downloaded gene lists of apoptosis, ferroptosis, immune, immunosuppression, and major histocompatibility complex (MHC) from GSEA (https://www.gsea-msigdb.org/gsea/downloads.jsp). We processed the data in R and presented the results as heatmaps as above.

### The Biological Significance of MXD3 Expression in Tumors

We used GSEA to explore the possible functional pathways of MXD3 in various tumors. We downloaded Gene Ontology (GO) and Kyoto Encyclopedia of Genes and Genomes (KEGG) gene sets from GSEA (https://www.gsea-msigdb.org/gsea/downloads.jsp). We used “clusterProfiler,” “enrichplot,” “ggplot2,” and other R packages to ascertain the GSEA function analysis process and draw the resulting graph. We selected cancers that had five or more significant functional pathways of MXD3 to generate the GSEA map.

### Analysis of Glioma of Different Grades

We analyzed different grades of glioma by the Chinese Glioma Genome Atlas (CGGA; http://www.cgga.org.cn/). We obtained the differential MXD3 and promoter in different grades and histological types of gliomas. We also generated primary glioma and recurrent glioma survival curves of the MXD3 and MXD3 promoters.

### Immunohistochemical Analysis of Glioma

We collected 25 pairs of vascular malformation (normal control) and glioma samples for immunohistochemistry. For IHC of glioma, following deparaffinization, hydration, and epitope retrieval, the activity of endogenous peroxidase in the slices was inhibited for 15 min by 3% hydrogen peroxide. Then, slides were incubated overnight at 4°C with an MXD3 primary antibody (1:200, Abcam, ab108525) in a humidified box and subsequently placed in secondary antibody. Finally, the slides were visualized by diaminobenzidine and counterstained with hematoxylin. Immunohistochemical sections were observed using an Olympus BX63 microscope, and the quantitative analysis of slides also used ImageJ software. Data were expressed as mean ± standard deviation. Statistical measurements were performed using SPSS 21.0 statistical software (SPSS Inc., Chicago, USA).

### Statistical Analysis

All gene mRNA expression data of cancers were standardized with log2 transformation. T-test or ANOVA was used to obtain the different data of groups. If there was no special statement, 0.05 was regarded as the cutoff point of significance. We used the Cox regression model, Kaplan–Meier method, and log-rank test for survival analysis. We used Spearman’s or Pearson’s test to determine the correlation between two genes. We accomplished all statistical processes in R software. Generally, three decimal places are reserved after the decimal point.

The abbreviations of TCGA cancer used in this paper and the corresponding full names are listed in Table 1.

**TABLE 1:** Cancer types from TCGA database.

Abbreviation	Full name
ACC	Adrenocortical carcinoma	
BLCA	Bladder urothelial carcinoma	
BRCA	Breast invasive carcinoma	
CESC	Cervical squamous cell carcinoma and endocervical adenocarcinoma	
CHOL	Cholangiocarcinoma	
COAD	Colon adenocarcinoma	
DLBC	Lymphoid neoplasm diffuse large B-cell lymphoma	
ESCA	Esophageal carcinoma	
GBM	Glioblastoma multiforme	
HNSC	Head and neck squamous cell carcinoma	
KICH	Kidney chromophobe	
KIRC	Kidney renal clear cell carcinoma	
KIRP	Kidney renal papillary cell carcinoma	
LAML	Acute myeloid leukemia	
LGG	Brain lower grade glioma	
LIHC	Liver hepatocellular carcinoma	
LUAD	Lung adenocarcinoma	
LUSC	Lung squamous cell carcinoma	
MESO	Mesothelioma	
OV	Ovarian serous cystadenocarcinoma	
PAAD	Pancreatic adenocarcinoma	
PCPG	Pheochromocytoma and paraganglioma	
PRAD	Prostate adenocarcinoma	
READ	Rectum adenocarcinoma	
SARC	Sarcoma	
SKCM	Skin cutaneous melanoma	
STAD	Stomach adenocarcinoma	
TGCT	Testicular germ cell tumor	
THCA	Thyroid carcinoma	
THYM	Thymoma	
UCEC	Uterine corpus endometrial carcinoma	
UCS	Uterine carcinosarcoma	
UVM	Uveal melanoma	

## Results

### MXD3 Had Significant Expression Differences Among Various Organs and Multiple Cancers

**FIGURE 1:**
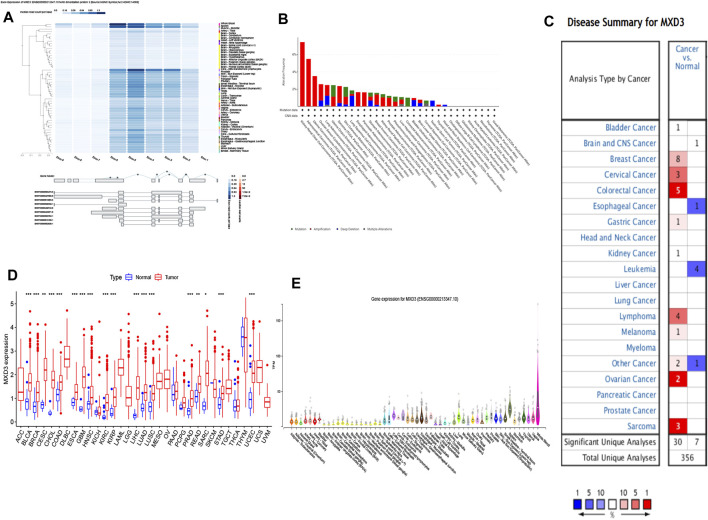
MXD3 expression and mutation of normal and cancer tissues: **(A)** expression of the MXD3 exon; **(B)** MXD3 mutation; **(C)** Oncomine dataset of cancer and paracancerous samples; **(D)** TCGA dataset of cancer and paracancerous samples; **(E)** different organs (***p < 0.001, **p < 0.01, *p < 0.05).

Among multiple organs, MXD3 had the highest expression in whole blood, followed by the spleen. MXD3 had a significantly lower expression in other organs, such as the skin, brain, heart, and liver (Figure 1E). The exon expression of MXD3 confirmed this conclusion and revealed that, in EBV-transformed lymphocytes, MXD3 also had high expression. In addition, exon 5 and exon 6 had the highest exon read counts per base among exons (Figure 1A). Using the cBioPortal tool, we found that the most common and predominant mutations in MXD3 were gene amplification, while gene deletions and mutations were less common (Figure 1B).

We compared both specimens based on TCGA dataset. We discovered that 18 classes of cancer had a vital discrepancy between the cancer sample and its paracancerous tissue. Cancer specimens had high expression levels versus paracancerous specimens in BLCA, BRCA, CESC, COAD, ESCA, GBM, HNSC, KIRC, KIRP, LIHC, LUAD, LUSC, PRAD, READ, SARC, STAD, and UCEC (Figure 1D). We also contrasted the cancer sample with its paracancerous tissue according to the Oncomine dataset, which showed that 13 types of cancer had differences (Figure 1C). Bladder cancer, breast cancer, cervical cancer, colorectal cancer, esophageal carcinoma, gastric cancer, kidney cancer, and sarcoma collectively had a discrepancy in both datasets. BLCA and BRCA showed the largest difference among the differentially expressed genes in both datasets.

### MXD3’s Prognostic Value Among Various Cancers

**FIGURE 2:**
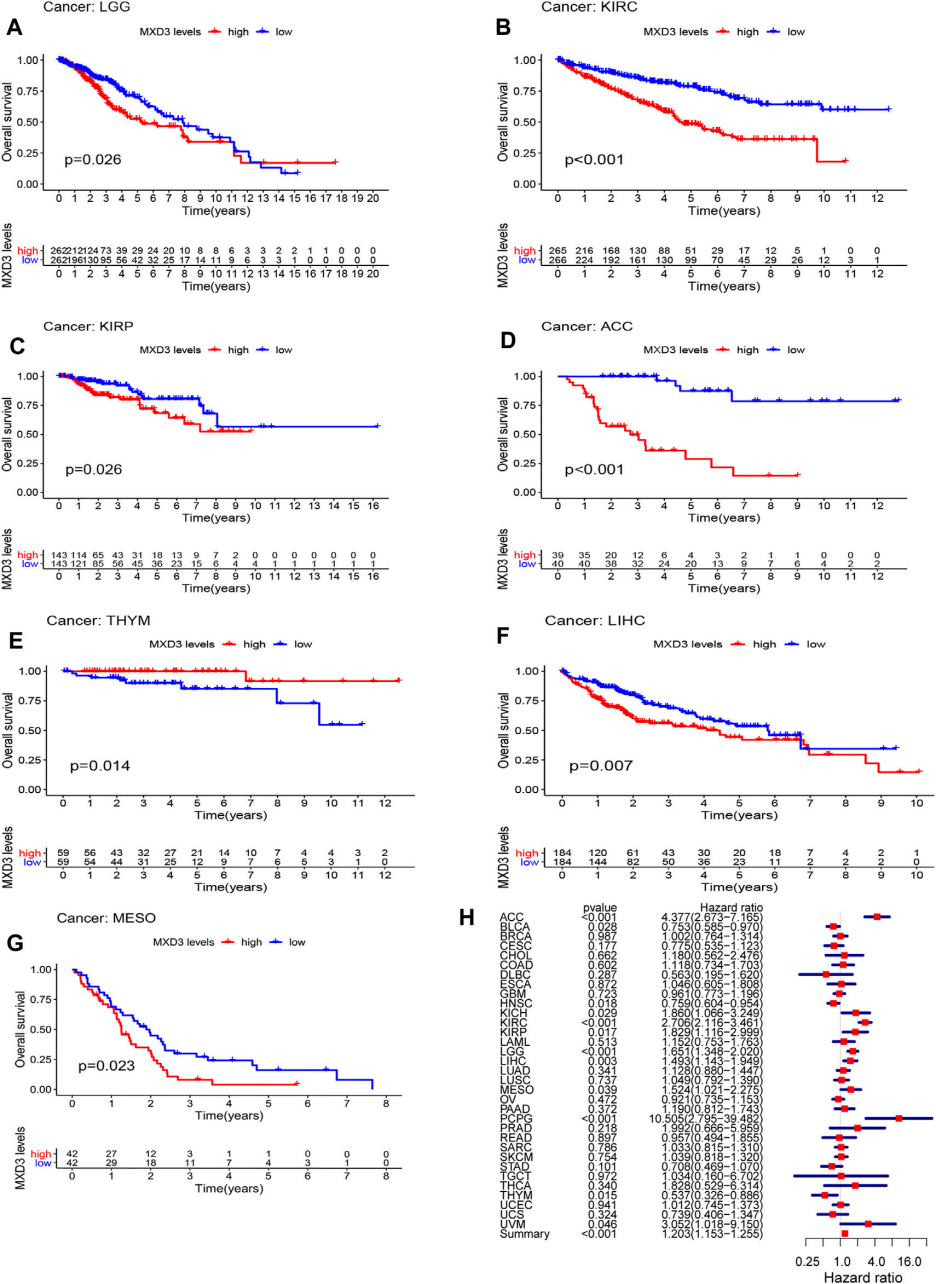
OS with different MXD3 expression in LGG **(A)**, KIRC **(B)**, KIRP **(C)**, ACC **(D)**, THYM **(E)**, LIHC **(F)**, and MESO **(G)**. Cox regression of MXD3 and OS **(H)** in cancer types.

To clarify the clinical value of MXD3, we analyzed the classic indexes of each cancer, such as OS, DSS, DFI, and PFI. Cox regression analysis was also performed. Patients suffering from LGG (Figure 2A, *p* = 0.026), KIRC (Figure 2B, *p* < 0.001), KIRP (Figure 2C, *p* = 0.026), ACC (Figure 2D, *p* < 0.001), THYM (Figure 2E, *p* = 0.014), LIHC (Figure 2F, *p* = 0.007), and MESO (Figure 2G, *p* = 0.023) with high MXD3 expression had worse OS according to Kaplan–Meier survival analysis. We used a univariate Cox regression model to determine the relationship between the expression level of MXD3 and OS. The results (Figure 2H) were as follows: KIRC (CI = [2.116, 3.461], *p* = 2.21E-15), ACC (CI = [2.673, 7.165], *p* = 4.38E-09), LGG (CI = [1.348, 2.020], *p* = 1.19E-06), PCPG (CI = [2.795, 39.481], *p* < 0.001), LIHC (CI = [1.142, 1.949], *p* = 0.003), THYM (CI = [0.326, 0.886], *p* = 0.015), KIRP (CI = [1.116, 2.998], *p* = 0.017), HNSC (CI = [0.604, 0.954], *p* = 0.018), BLCA (CI = [0.585, 0.970], *p* = 0.028), MESO (CI = [1.021, 2.275], *p* = 0.039), UVM (CI = [1.018, 9.150], *p* = 0.046), and summary (CI = [1.153, 1.225], *p* < 0.001).

**FIGURE 3:**
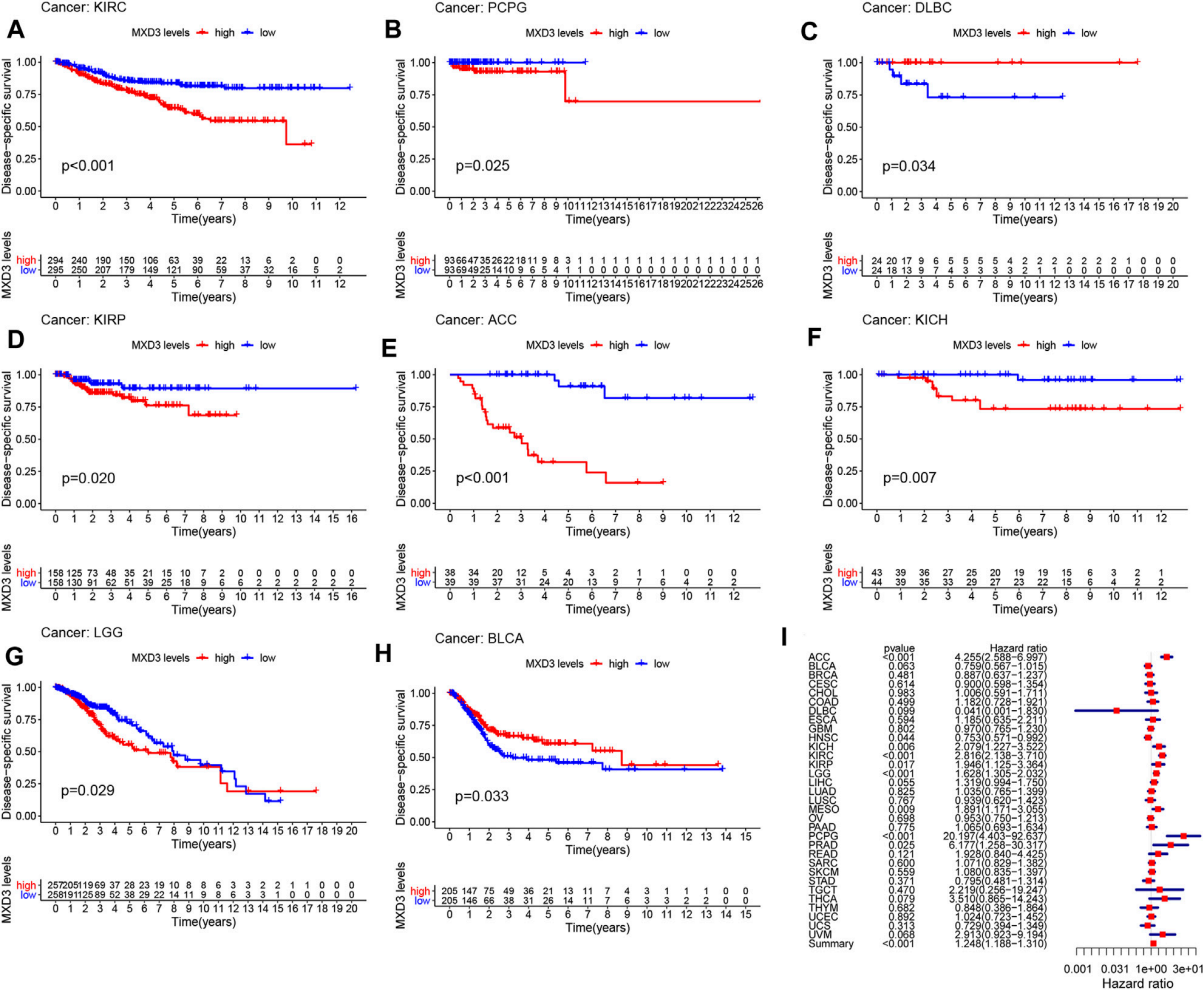
DSS with different MXD3 expression in KIRC **(A)**, PCPG **(B)**, DLBC **(C)**, KIRP **(D)**, ACC **(E)**, KICH **(F)**, LGG **(G)**, and BLCA **(H)**. Cox regression of MXD3 and DSS **(I)** in cancer types.

Furthermore, the DSS of patients suffering from KIRC (Figure 3A, *p* < 0.001), PCPG (Figure 3B, *p* = 0.025), DLBC (Figure 3C, *p* = 0.034), KIRP (Figure 3D, *p* = 0.020), ACC (Figure 3E, *p* < 0.001), KICH (Figure 3F, *p* = 0.007), LGG (Figure 3G, *p* = 0.029), and BLCA (Figure 3H, *p* = 0.033) with high MXD3 expression was lower than that of patients with low expression. The expression level of MXD3 was associated with DSS (Figure 3I) in ACC (CI = [2.586, 6.997], *p* = 1.16E-08), KIRC (CI = [2.138, 3.710], *p* = 1.82E-13), LGG (CI = [1.305, 2.032], *p* = 1.59E-05), PCPG (CI = [4.403, 92.637], *p* < 0.001), KICH (CI = [1.227, 3.522], *p* = 0.006), MESO (CI = [1.171, 3.055], *p* = 0.009), KIRP (CI = [1.125, 3.362], *p* = 0.0.017), HNSC (CI = [0.571, 0.992], *p* = 0.044), PRAD (CI = [1.258, 30.317], *p* = 0.025), and summary (CI = [1.188, 1.310], *p* < 0.001) according to the univariate Cox regression model.

**FIGURE 4:**
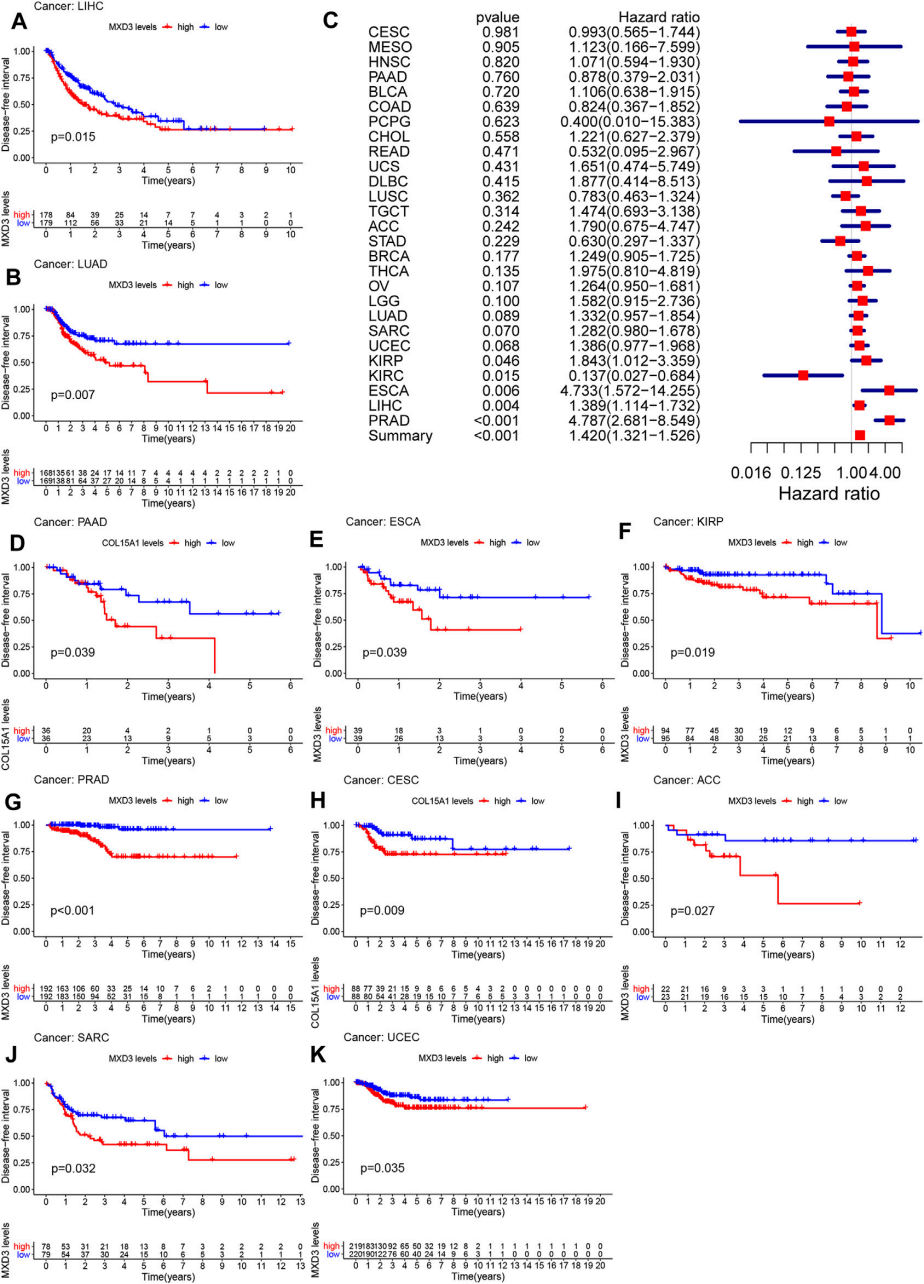
DFI with different MXD3 expression in LIHC **(A)**, LUAD **(B)**, PAAD **(D)**, ESCA **(E)**, KIRP **(F)**, PRAD **(G)**, CESC **(H)**, ACC **(I)**, SARC **(J)**, and UCEC **(K)**. Cox regression of MXD3 and DFI **(C)** in cancer types.

The DFI of patients suffering from LIHC (Figure 4A, *p* = 0.015), LUAD (Figure 4B, *p* = 0.007), PAAD (Figure 4D, *p* < 0.001), ESCA (Figure 4E, *p* = 0.009), KIRP (Figure 4F, *p* = 0.019), PRAD (Figure 4G, *p* < 0.001), CESC (Figure 4H, *p* = 0.009), ACC (Figure 4I, *p* = 0.027), SARC (Figure 4J, *p* = 0.032), and UCEC (Figure 4K, *p* = 0.035) with high MXD3 expression was higher than that of patients with low expression. The expression level of MXD3 was associated with DSS (Figure 4C) in ESCA (CI = [1.572, 14.255], *p* = 0.06), KIRC (CI = [0.027, 0.684], *p* = 0.015), KIRP (CI = [1.012, 3.359], *p* = 0.046), LIHC (CI = [1.114, 1.732], *p* = 0.004), PRAD (CI = [2.681, 8.549], *p* < 0.001), and summary (CI = [1.321, 1.526], *p* < 0.001) according to the univariate Cox regression model.

**FIGURE 5:**
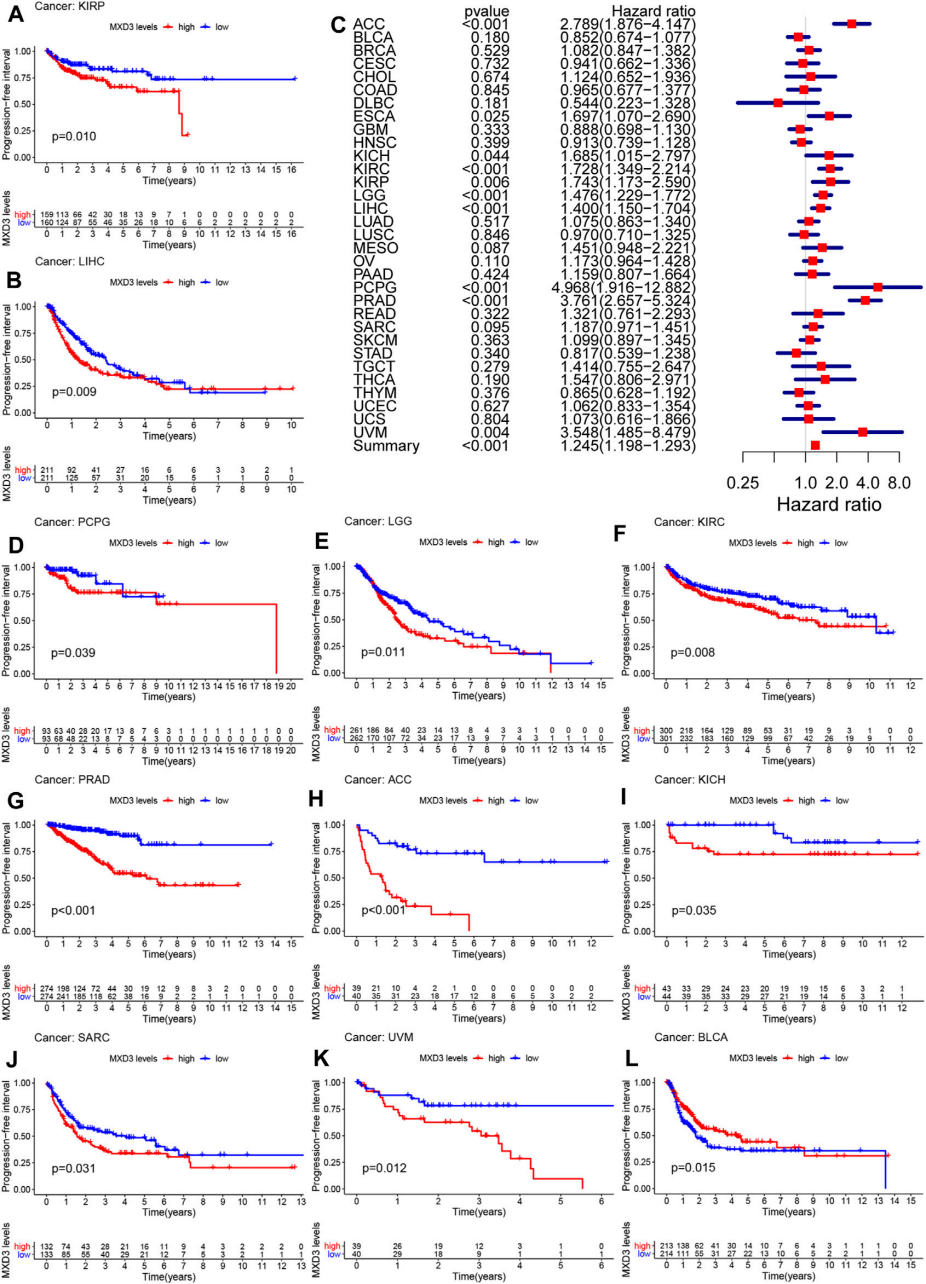
PFI with different MXD3 expression in KIRP **(A)**, LIHC **(B)**, PCPG **(D)**, LGG **(E)**, KIRC **(F)**, PRAD **(G)**, ACC **(H)**, KICH **(I)**, SARC **(J)**, UVM **(K)**, and BLCA**(L)**. Cox regression of MXD3 and PFI **(C)** in cancer types.

The PFI of patients suffering from KIRP (Figure 5A, *p* = 0.010), LIHC (Figure 5B, *p* = 0.009), PCPG (Figure 5D, *p* = 0.039), LGG (Figure 5E, *p* = 0.011), KIRC (Figure 5F, *p* = 0.008), PRAD (Figure 5G, *p* < 0.001), ACC (Figure 5H, *p* < 0.001), KICH (Figure 5I, *p* = 0.035), SARC (Figure 5J, *p* = 0.031), UVM (Figure 5K, *p* = 0.012), and BLCA (Figure 5L, *p* = 0.015) with higher MXD3 expression was longer than that of patients with lower expression. The expression level of MXD3 was associated with DSS (Figure 5C) in ACC (CI = [1.876, 4.147], *p* < 0.001), ESCA (CI = [1.070, 2.690], *p* = 0.025), KICH (CI = [1.012, 3.359], *p* = 0.044), KIRC (CI = [1.349, 2.214], *p* < 0.001), KIRP (CI = [1.173, 2.590], *p* = 0.004), LGG (CI = [1.229, 1.772], *p* < 0.001), LIHC (CI = [1.150, 1.704], *p* < 0.001), PCPG (CI = [1.916, 12.882], *p* < 0.001), PRAD (CI = [2.657, 5.324], *p* < 0.001), UVM (CI = [1.485, 8.479], *p* = 0.004), and summary (CI = [1.198, 1.293], *p* < 0.001) according to the univariate Cox regression model.

**FIGURE 6:**
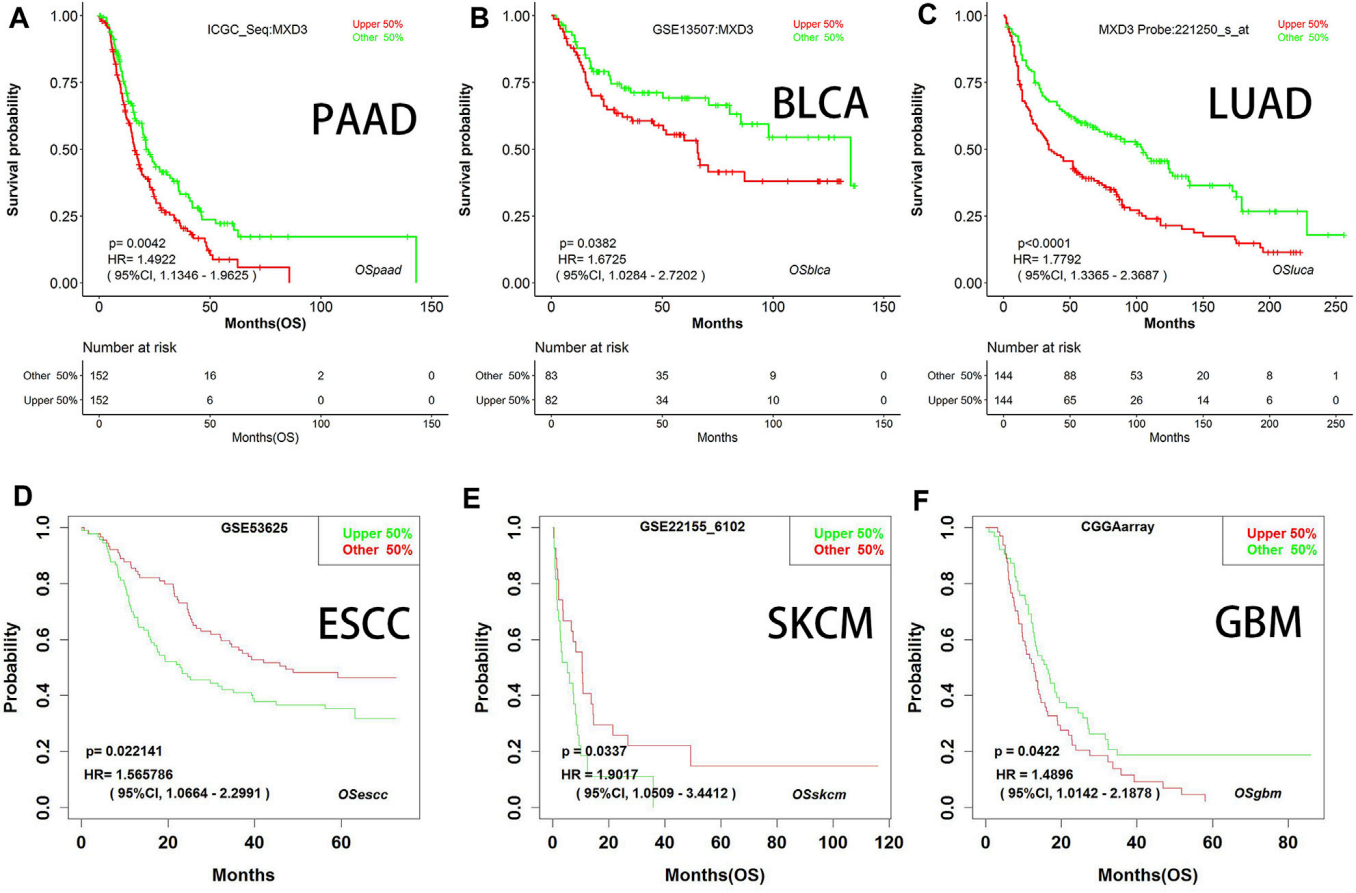
OS with different MXD3 expression **(A–F)** in GEO in PAAD **(A)**, BLCA **(B)**, LUAD **(C)**, ESCA **(D)**, SKCM **(E)**, and GBM **(F)**.

We generated survival curves for different types of cancers in the GEO dataset through BII, a web tool. Patients with high MXD3 expression had poorer survival than patients with low MXD3 expression in PAAD (Figure 6A; *p* = 0.004), BLCA (Figure 6B; *p* = 0.038), LUAD (Figure 6C; *p* < 0.0001), ESCA (Figure 6D; *p* = 0.024), SKCM (Figure 6E; *p* = 0.034), and GBM (Figure 6F; all *p* = 0.042). Furthermore, we identified the prognostic indicators of MXD3 in other GEO datasets using the PrognoScan web tool. UVM, at the optimal cutoff point, LGG, DLBC, and OV patients with a higher expression of MXD3 had worse OS prognosis, while UVM patients with a higher expression of MXD3 had better prognosis (Supplementary Table S1).

Besides, we also validated the relationship between the differential expression of MXD3 and the patient outcome within the ICI cohort. Bladder cancer patients with high MXD3 expression had a better prognosis after treatment with ICIs (Supplementary Figure S6A; *p* = 0.005).

### Relationship Between MXD3 Methylation and OS in Multiple Cancers

**FIGURE 7:**
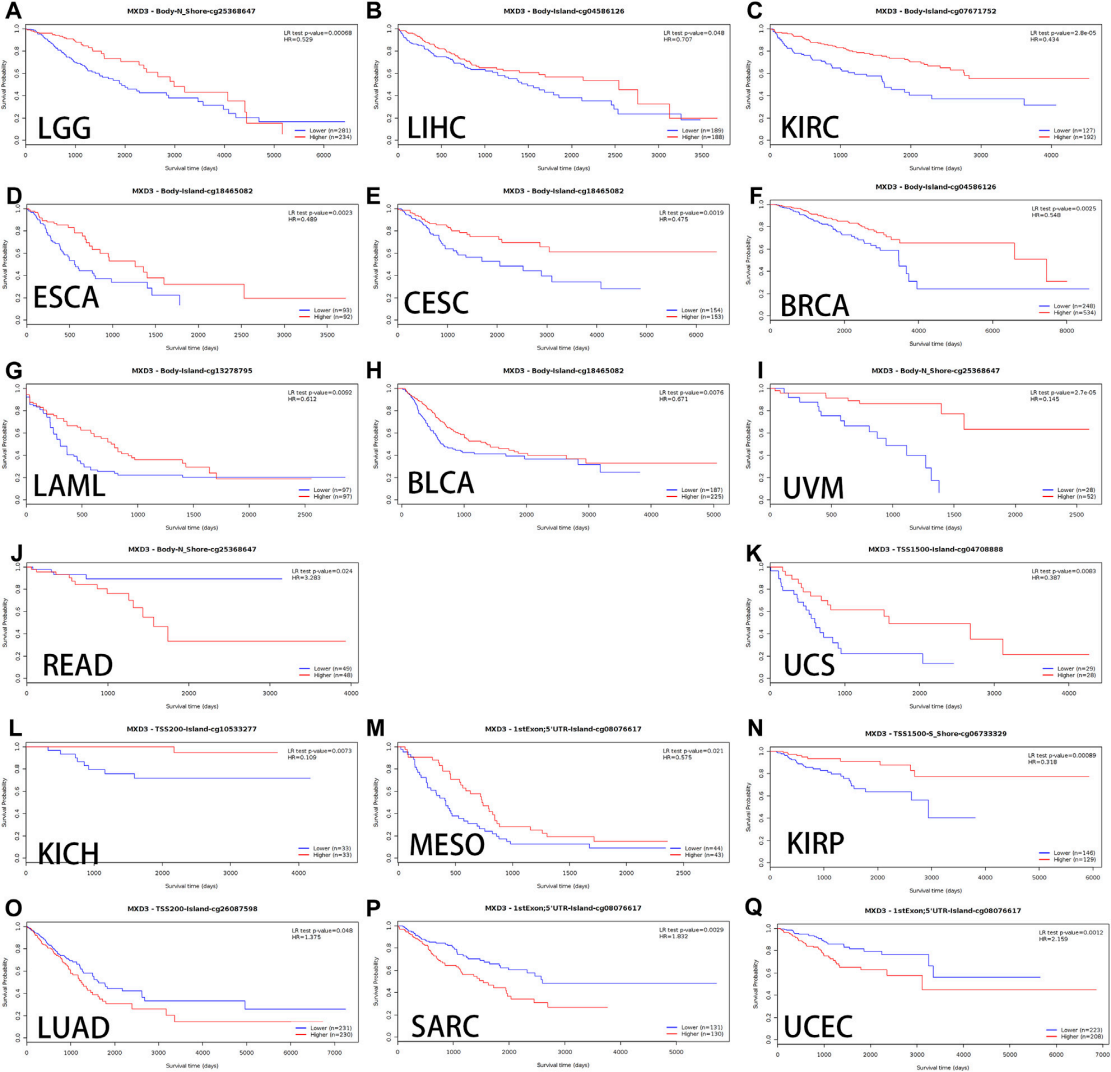
OS of different MXD3 gene bodies in LGG **(A)**, LIHC **(B)**, KIRC **(C)**, LAML **(G)**, UVM **(I)**, BLCA **(H)**, CESC **(E)**, ESCA **(D)**, BRCA **(F)**, and READ **(J)**. OS of different promoter methylation statuses in KICH **(L)**, UCS **(K)**, KIRP **(N)**, MESO **(M)**, SARC **(P)**, LUAD **(O)**, and UCEC **(Q)**.

Alterations in DNA methylation are common in various tumors as well as during development. Hypomethylation, which inhibits transcription of tumor suppressor gene promoter regions leading to gene silencing, has been most extensively studied among all types of epigenetic modifications (Das and Singal, 2004a). Genomic methylation might be an unexpected therapeutic target for DNA methylation inhibitors, leading to the normalization of gene overexpression induced during carcinogenesis (Yang et al., 2014a). We used MethSurv to analyze the methylation data of TCGA. The cancers with better OS curves for MXD3 body hypermethylated expression were as follows: LGG (Figure 7A, *p* < 0.001), LIHC (Figure 7B, *p* = 0.048), KIRC (Figure 7C, *p* < 0.001), LAML (Figure 7G, *p* = 0.009), UVM (Figure 7I, *p* < 0.001), BLCA (Figure 7H, *p* = 0.008), CESC (Figure 7E, *p* = 0.002), BRCA (Figure 7F, *p* = 0.003), and ESCA (Figure 7D, *p* = 0.002). In READ (Figure 7J, *p* < 0.024), MXD3 body hypermethylated expression had a poor prognosis. Patients with MXD3 promoter hypermethylation had better OS in KICH (Figure 7L, *p* = 0.007), UCS (Figure 7K, *p* = 0.008), KIRP (Figure 7N, *p* < 0.001), and MESO (Figure 7M, *p* = 0.021). Conversely, patients with the same methylation status had worse OS in SARC (Figure 7P, *p* = 0.003), LUAD (Figure 7O, *p* = 0.048), and UCEC (Figure 7Q, *p* = 0.001).

### Differential Expression of MXD3 Between Different Clinical Manifestations in Various Cancers

**FIGURE 8:**
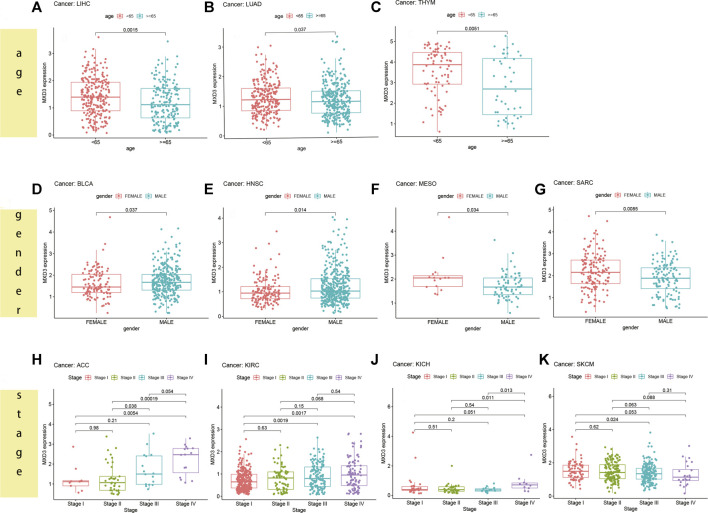
MXD3 expression of different clinical phenotypes of age in LIHC **(A)**, LUAD **(B)**, and THYM **(C)**. MXD3 expression of different clinical phenotypes of gender in MESO **(F)**, SARC **(G)**, BLCA **(D)**, and HNSC **(E)**. MXD3 expression of different clinical phenotypes of stage in ACC **(H)**, KIRC **(I)**, KICH **(J)**, and SKCM **(K)**.

For patients suffering from LIHC (Figure 8A, *p* = 0.015), LUAD (Figure 8B, *p* = 0.037), and THYM (Figure 8C, *p* = 0.005), the MXD3 expression level was higher in younger patients (<65) than in older patients (≥65). However, in most cancers, there was no significant difference in the expression of MXD3 between the two age groups (Supplementary Figure S1). Female patients suffering from MESO (Figure 8F, *p* = 0.034) and SARC (Figure 8G, *p* = 0.009) had higher MXD3 expression than males, but female patients suffering from BLCA (Figure 8D, *p* = 0.037) and HNSC (Figure 8E, *p* = 0.014) had lower expression. In the remaining cancers, there was no significant difference in the expression of MXD3 between the sexes (Supplementary Figure S2). Patients suffering from ACC (Figure 8H, *p* = 0.005), KIRC (Figure 8I, *p* = 0.002), KICH (Figure 8J, *p* = 0.051), and SKCM (Figure 8K, *p* = 0.053) with stage IV versus stage I had higher MXD3 expression. Patients suffering from ACC (*p* < 0.001) and KICH (*p* = 0.001) with stage IV versus stage II had higher MXD3 expression. In most of the remaining cancers, the expression of MXD3 was not significantly different between different tumor stages (Supplementary Figure S3).

### Correlations of MXD3 Expression Levels With Tumor Mutation Burden and Tumor Microsatellite Instability

**FIGURE 9:**
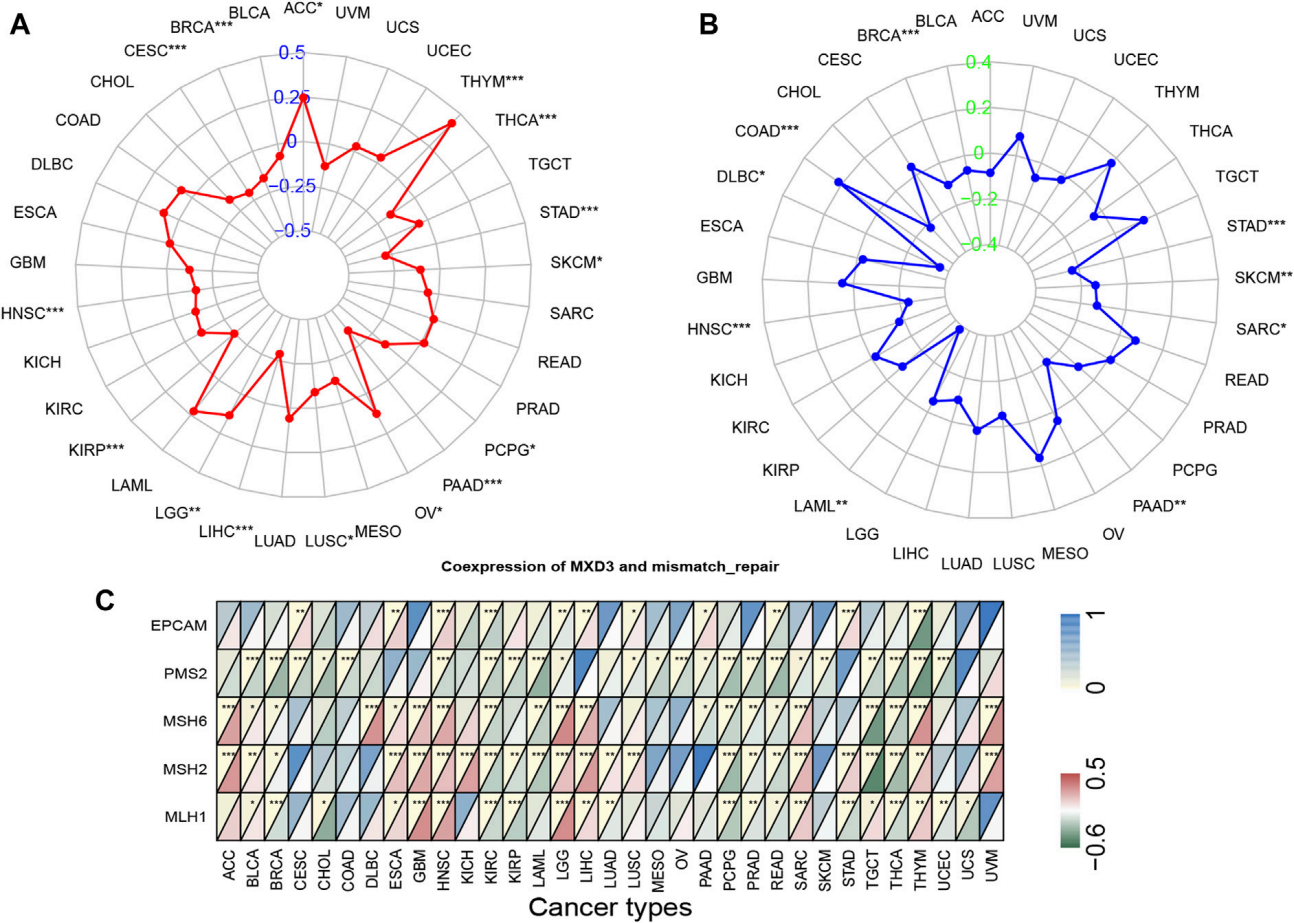
TMB **(A)**, MSI **(B)**, and mismatch repair genes **(C)** correlated with MXD3 expression.

In 33 types of cancer, we tested the relationship between gene expression levels and MSI and TMB, which are both beneficial for clinical immunotherapy (Das and Singal, 2004a). The outcome of TMB analysis indicated that MXD3 was correlated with TMB in STAD, LIHC, BRCA, and 12 other cancers (Figure 9A). Additionally, we determined that MXD3 was correlated with MSI in STAD, HNSC, COAD, and six other cancers (Figure 9B). Regardless of tissue cancer, numerous mutant neoantigens in mismatch repair–deficient cancers (Figure 9C) make them sensitive to immune checkpoint blockade (Le et al., 2017). MXD3 was positively correlated with mismatch repair genes overall in various cancers. In GBM and HNSC, MXD3 expression was identified more closely with mismatch repair genes. Using the web tool TISIDB, we obtained the MXD3-related expression profile of immunotherapy (Supplementary Table S2). The log-fold change of the immunotherapy response group vs. the immunotherapy non-response group was 0.215 in urothelial cancer. However, there was no significant difference in MXD3 expression between the two groups in other cancers.

### Correlation Between the Tumor Microenvironment and the Expression of MXD3

**FIGURE 10:**
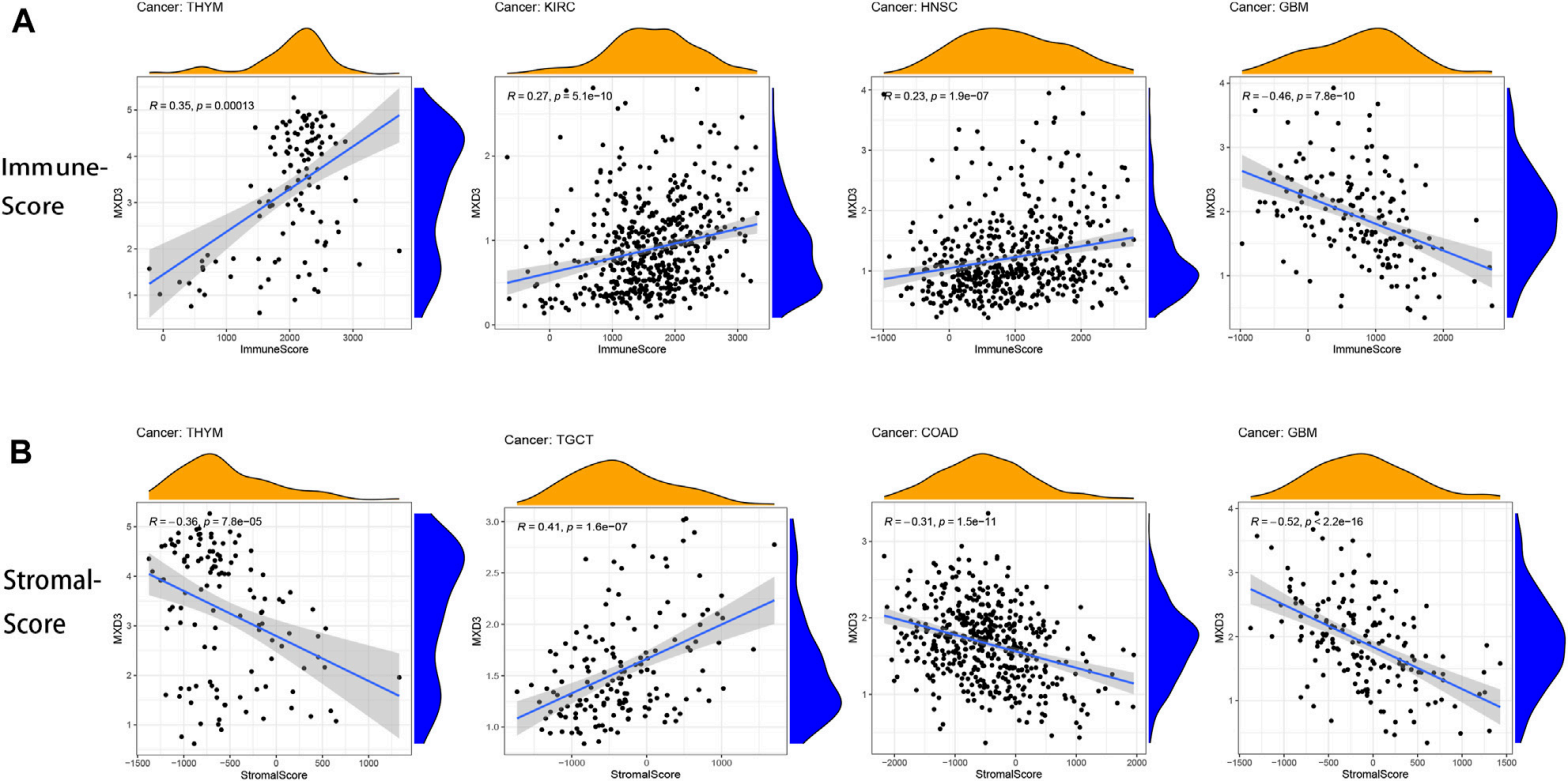
**(A)** The four cancers with the highest correlation between the immune score and MXD3 expression. **(B)** The four cancers with the highest correlation between the stromal score and MXD3 expression.

The tumor immune microenvironment is both a consequence and a cause of cancer development and an important factor in maintaining cancer cell growth (Goodman et al., 2019). The correlation of immune score and MXD3 expression revealed a close connection between THYM, KIRC, HNSC, and GBM (Figure 10A; all *p* < 0.001). The connection between the stromal score and MXD3 expression was considerably close in THYM, COAD, TGCT, and GBM (Figure 10B; all *p* < 0.001).

### Relationship Between Immune Infiltrating Cells and the Expression of MXD3

**FIGURE 11:**
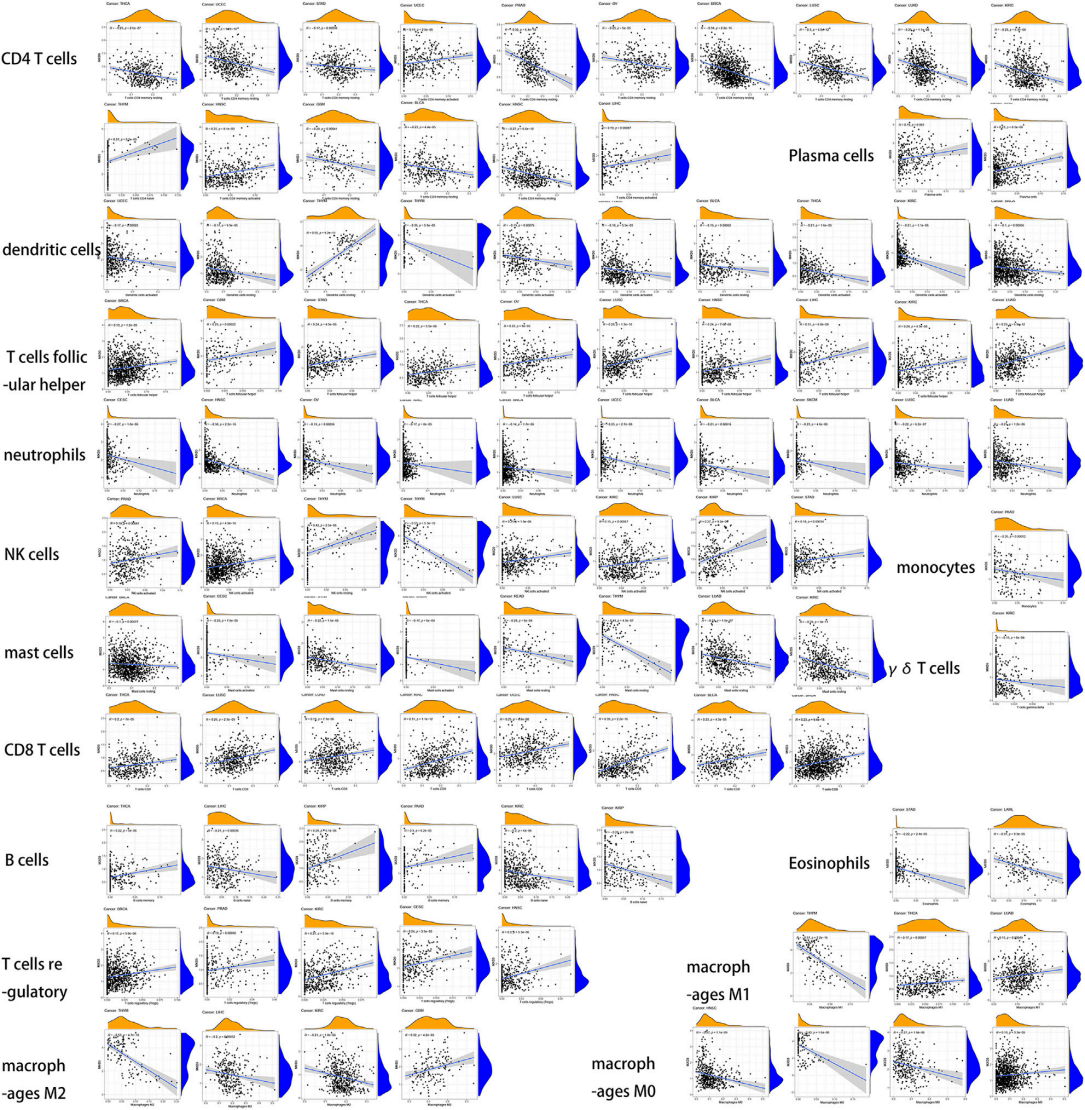
Dependence analysis of MXD3 expression on infiltrating immune cells in different types of cancers.

We then analyzed the infiltration of immune cells in the tumor and combined it with the MXD3 expression level (Figure 11). Our analysis showed that MXD3 in CD4 T cells had clear relationships with nearly half of tumors. In most tumors, the numbers of CD4 T cells were negatively correlated with MXD3 expression. Similarly, in most of the cancers that had a significant relationship with MXD3 expression in dendritic cells, a type of antigen-presenting cell, the number of dendritic cells was negatively correlated with MXD3 expression. The numbers of follicular T cells and CD8 T helper cells were significantly positively correlated with MXD3 expression in tumors. The numbers of neutrophils and mast cells were significantly negatively correlated with MXD3 expression in tumors. The number of NK cells was significantly positively correlated with MXD3 expression in almost all tumors.

### Coexpression of MXD3 and Immune, Apoptosis, and Ferroptosis Pathway–Related Genes

**FIGURE 12:**
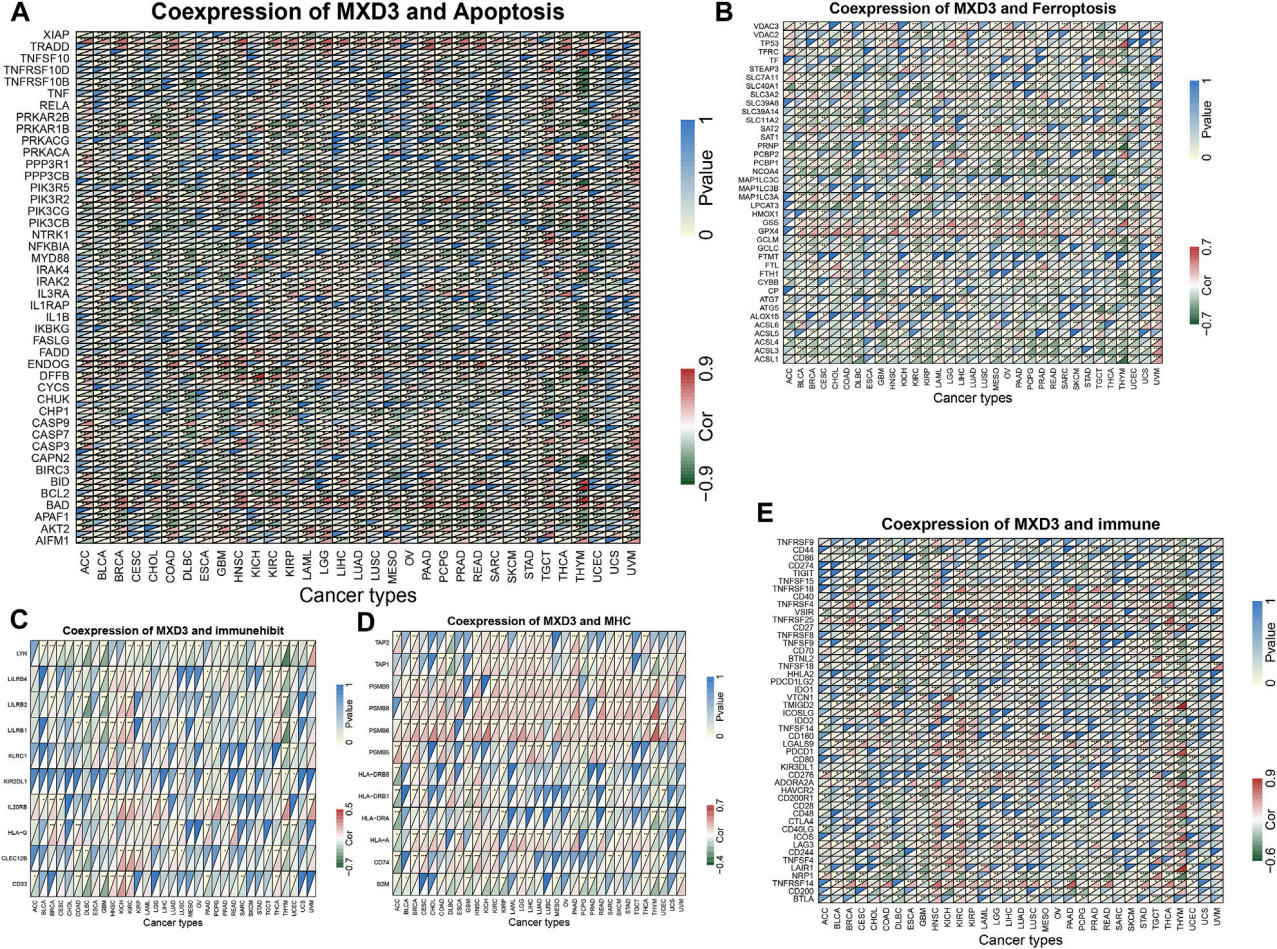
Coexpression of MXD3 with apoptosis **(A)**, ferroptosis **(B)**, immunosuppression **(C)**, MHC **(D)**, and immune **(E)** gene sets.

In all cancers, MXD3 is associated with most apoptosis pathway–related genes (Figure 12A), confirming previous reports that MXD3 knockdown can induce apoptosis (Barisone et al., 2015; Duong et al., 2017). Except for USC, ACC, and ESCA, MXD3 in most cancers was closely related to ferroptosis (Figure 12B). The coexpression relationship between genes involved in immunosuppression pathways (Figure 12C) and MXD3 was not considerably significant, and only a few genes in a small number of tumors showed a significantly strong correlation with MXD3. PSMB6, PSMB8, and PSMB9, all genes of the MHC pathway (Figure 12D), had vital positive coexpression with MXD3. In general, MXD3 was positively coexpressed with genes of the immune-related pathway (Figure 12E) across HNSC, KIRC, and THCA.

### KEGG Analysis and GO Analysis of MXD3 Expression Levels From GSEA

To identify the pathways in which genes act in cancers, we performed GSEA and GO analysis of the MXD3 expression level, taking the intersection of the two analyses with more than five significant pathways.

**FIGURE 13:**
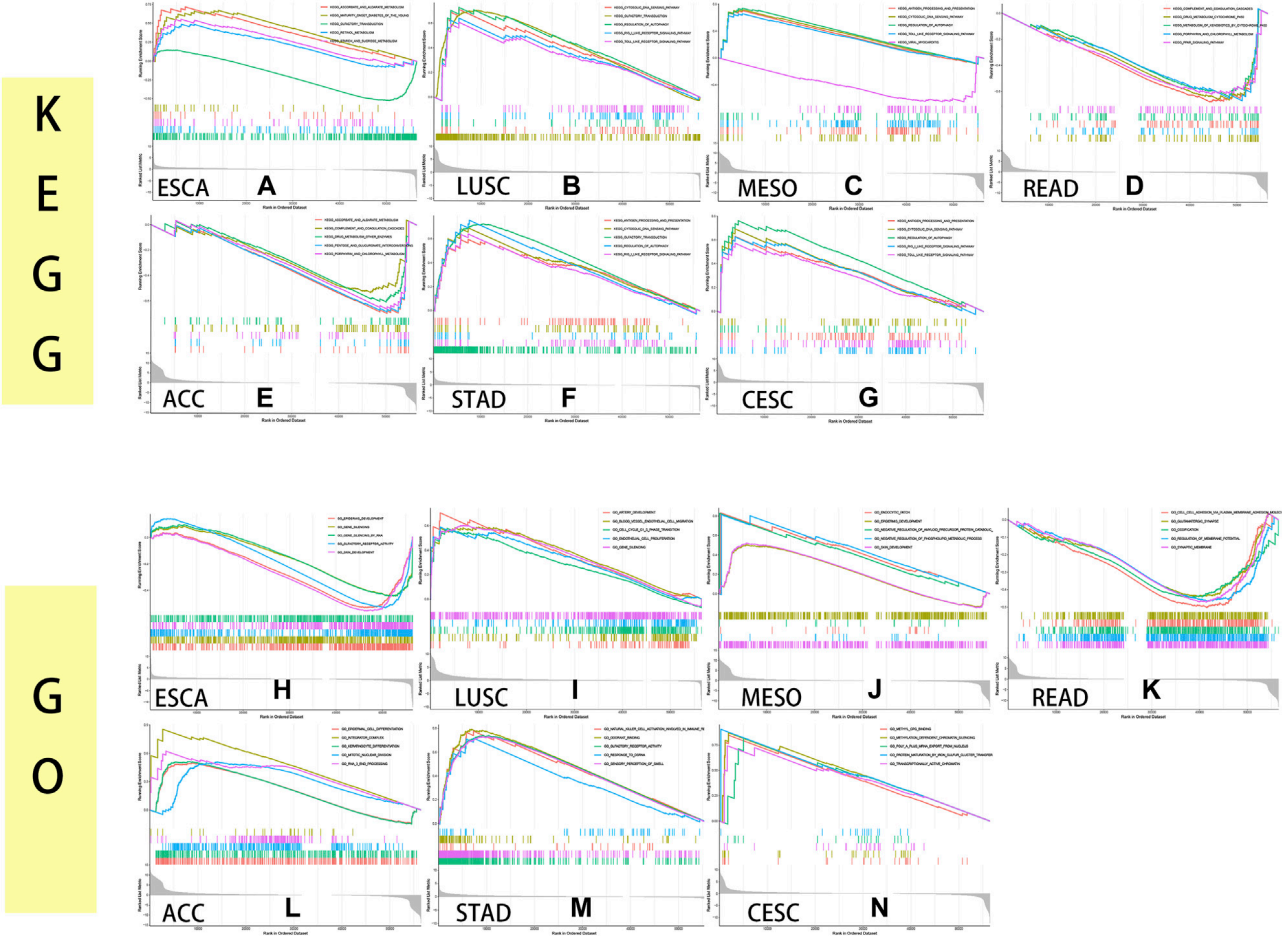
KEGG and GO GSEA of MXD3 in ESCA **(A, H)**, LUSC **(B, I)**, MESO **(C, J)**, READ **(D, K)**, ACC **(E, L)**, STAD **(F, M)**, and CESC **(G, N)**.

The KEGG analysis showed that MXD3 expression was positively correlated with the regulation of the autophagy pathway and cytosolic DNA sensing pathway in LUSC (Figure 13B), STAD (Figure 13F), CESC (Figure 13G), and MESO (Figure 13C). Similarly, MXD3 expression was positively correlated with the RIG-I-like receptor signaling pathway in LUSC, STAD, and CESC and related to the Toll-like receptor signaling pathway in LUSC, MESO, and CESC. The antigen processing and presentation pathway was positively related to MXD3 in MESO, STAD, and CESC. Intriguingly, MXD3 expression was positively related to olfactory transduction in LUSC and STAD but negatively related to olfactory transduction in ESCA (Figure 13A). MXD3 expression was negatively related to the complement and coagulation cascades signaling pathway in READ (Figure 13D) and ACC (Figure 13E).

GO analysis indicated that the selected GO pathways were quite different among the selected cancers. MXD3 expression was positively correlated with epidermal development in MESO (Figure 13J) and ESCA (Figure 13H) and related to skin development in ESCA and MESO. Similarly, MXD3 expression was positively correlated with olfactory receptor activity in ESCA and STAD (Figure 13M). In LUSC (Figure 13I), MXD3 expression was positively related to cell cycle G1_S phase transition. MXD3 expression was negatively related to glutamatergic synapse in READ (Figure 13K). In ACC (Figure 13L), MXD3 expression was positively related to the integrator complex. In CESC (Figure 13N), MXD3 expression was positively related to methylation-dependent silencing and methyl-CpG binding. The GO analysis results of the remaining 25 cancers are shown in Supplementary Figure S5.

### Differences in MXD3 Expression and OS of MXD3 at Different Expression Levels in Different Grades of Glioma

**FIGURE 14:**
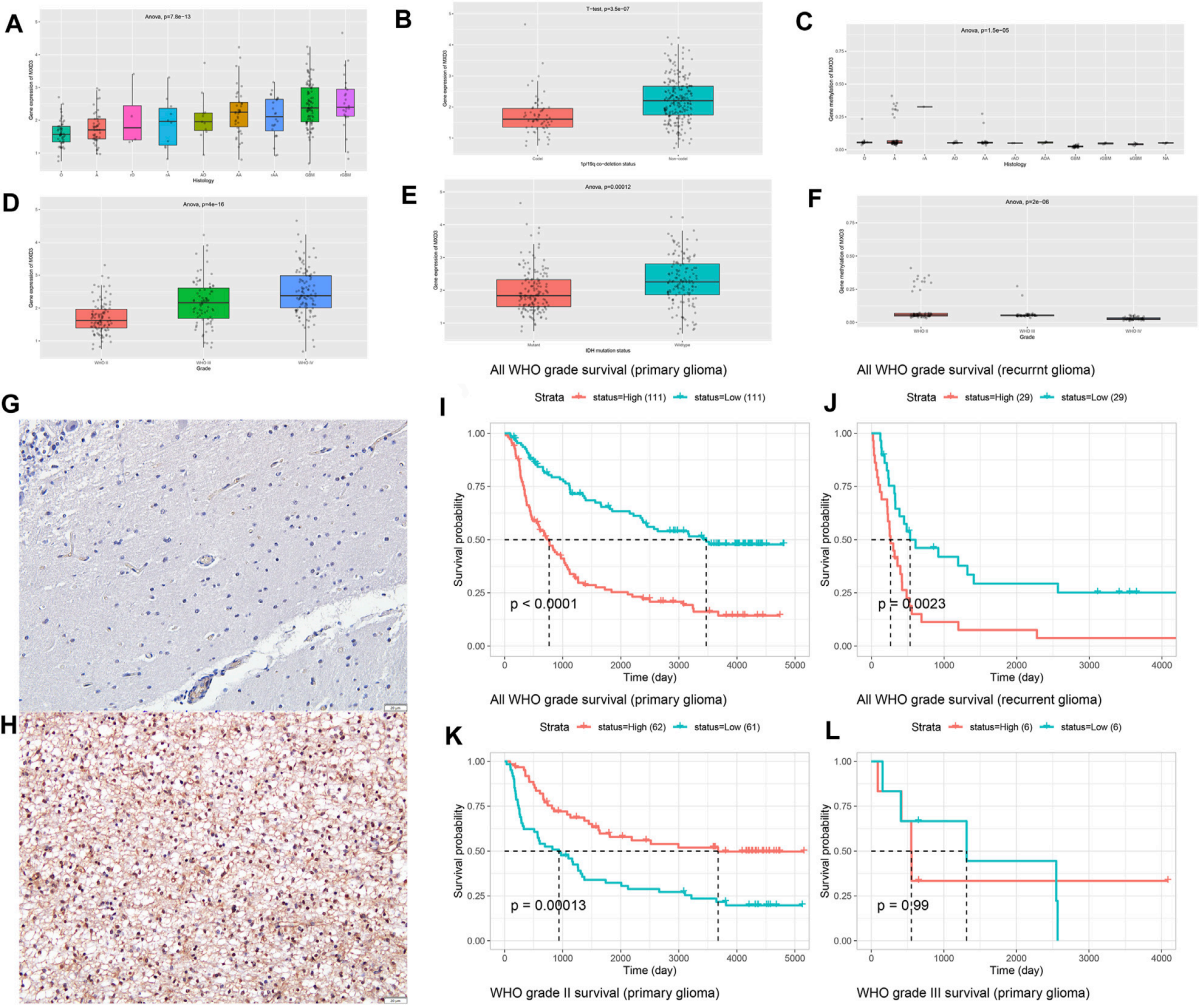
Differential expression of MXD3 in tissues **(A)** and WHO subtypes **(D)** of glioma. Differential expression of MXD3 in 1p/19q codeletion **(B)** and IDH mutation status **(E)**. Differential expression of MXD3 promoter methylation in tissues **(C)** and WHO subtypes **(F)** of glioma. Representative images of MXD3 immunohistochemistry in glioma. The image shows a 16x magnification. Scale bars = 50 μm (normal **(G)**, glioma **(H)**. OS of MXD3 expression in primary glioma **(I)** and recurrent glioma **(J)**. OS of MXD3 promoter methylation status in primary glioma **(K)** and recurrent glioma **(L)**.

Different histological types of patients corresponded to different expression levels of MXD3 (Figure 14A; *p* = 7.8e -13). With the increase of malignancy (astrocytoma (A), recurrent astrocytoma (RA), anaplastic astrocytoma (AA), recurrent anaplastic astrocytoma (rAA), oligodendroglioma (O), recurrent oligodendroglioma (rO), anaplastic oligodendroglioma (AO), glioblastoma (GBM), and recurrent glioblastoma (rGBM)), the expression of MXD3 increased significantly. With the increase in WHO classification, the expression of MXD3 also increased (Figure 14D; P = 4e -16). The expression of MXD3 was also significantly increased in patients with IDH mutation and/or codeletion of 1p19q (Figure 14B, *p* = 3.5e-7; Figure 14E, *p* < 0.001). OS was significantly different between patients with either primary or recurrent gliomas (Figure 14I, *p* < 0.0001; Figure 14J, *p* = 0.002).

The promoter methylation of MXD3 was statistically significant (Figure 14C; *p* = 1.5e-5) but not significantly different in patients with each degree of pathology. Similarly, there were significant differences in the promoter methylation of MXD3 among the different WHO glioma subtypes (Figure 14F; *p* = 2e-6). For gliomas of primary origin, lower MXD3 promoter methylation indicated worse patient outcomes (Figure 14K, *p* < 0.001). However, the difference in MXD3 promoter methylation across recurrent glioma patients was not significant (Figure 14L, *p* = 0.99).

To determine MXD3 expression in cancers, we chose glioma as the target cancer because its high expression in glioma implies meaningful poor survival in both TCGA and GEO datasets. We used hemangioma samples as controls (*n* = 25). IHC results showed that the nuclear localization of the MXD3 protein was significantly increased in tumor samples (Figure 14H) compared to normal tissue (Figure 14G). The cell counting of immunohistochemistry in this typical image was 72.54%.

## Discussion

Our study suggests that 18 types of cancer have higher MXD3 expression in tumor samples. MXD3 is a proliferation-promoting factor in neuroblastoma and B-cell lymphoma (Barisone et al., 2012; Barisone et al., 2015), which is consistent with our analysis. Our conclusion was verified by the Oncomine dataset, which is consistent with the results of a study on granular neuron precursors (Yun et al., 2007) that confirmed the proliferative effect of MXD3. MXD3 may be regulated by E2F1 as a transcriptional repressor gene (Fox and Wright, 2003).

TCGA data from Kaplan–Meier survival analysis suggested that high MXD3 expression indicates a poor prognosis for patients suffering from ACC, LIHC, KIRC, LGG, MESO, and KIRP. Patients suffering from BLCA, LUAD, ESCA, SKCM, LGG, DLBC, GBM, and OV with high MXD3 expression had a poor prognosis based on the GEO dataset. These findings suggest that MXD3 is an indicator of poor prognosis in a variety of cancers, especially gliomas, which is why we chose glioma for verification.

The prognosis of patients with high MXD3 bladder cancer in the GEO database is poor, but the bladder patients in the MXD3 group have better prognosis after treatment with the immunotherapeutic point inhibitor. It is suggested that patients with high MXD3 expression may have more benefits from ICI treatment.

For patients suffering from LIHC, LUAD, and THYM, the MXD3 expression level was higher in younger patients than in older patients. This finding might suggest age grouping of different cancers for targeted therapies. There was no significant difference in the expression of MXD3 in different cancer types between male and female patients, suggesting that there was no significant relationship between gene expression levels and sex. Patients suffering from ACC, KIRC, and KICH with stage IV versus stage I had higher MXD3 expression. These results might indicate that high MXD3 expression is suggestive of a high-grade tumor stage and may help determine patient prognosis.

Hypermethylation of the gene body is significantly and positively correlated with gene expression (Yang et al., 2014b; Seymour and Gaut, 2020). In contrast, gene promoter hypermethylation can silence current genes (Das and Singal, 2004b). In our study, the majority of individuals with hypermethylated MXD3 bodies had a better prognosis, whereas the prognosis of patients with promoter hypermethylation was complicated. This fact may be due to the relationship between the MYC pathway and the gene methylation pathway (Das and Singal, 2004a). Nevertheless, our study confirmed the role of MXD3 methylation in predicting prognosis, which plays an important role in guiding the use of the methyltransferase inhibitor decitabine and other drugs.

There is a clear correlation between high TMB and improved survival in a variety of cancer tissues, but the cutoff point varies by tissue (Samstein et al., 2019). Furthermore, TMB and not just high TMB improves patient outcomes in patients treated with immune checkpoint inhibitors (ICIs) (Samstein et al., 2019). The relationship between high TMB and ICI treatment has also been demonstrated in lung, esophageal, and triple-negative breast cancers (Fang et al., 2019; Greally et al., 2019; Barroso-Sousa et al., 2020). Previous studies with large samples of pancarcinomas have suggested that overall MSI is associated with patient outcomes (Hause et al., 2016). Overall, MXD3 expression was negatively correlated with TMB in 15 cancers and MSI in 9 cancers. These results might suggest that MXD3 expression levels will affect TMB and MSI in cancers and influence the patient response to immune checkpoint inhibition therapy. These findings provide new references for the prognosis of immunotherapy. We also found that MXD3 expression was positively correlated with MMR gene expression in most tumors. Based on the existing studies and our findings, low MXD3 expression seems to influence the efficacy of ICI therapy for tumors.

In gallbladder and gastric cancers, the TME is an indicator of prognosis and sensitivity to ICI treatment (Zeng et al., 2019; Cao et al., 2021). We performed immune and stromal scoring with ESTIMATE. Overall, MXD3 was positively correlated with the immune score in most cancers but negatively correlated with the stromal score. Immune cells within the tumor microenvironment (TME) play an important role in tumorigenesis. These tumor-associated immune cells have either antitumor or tumor-promoting functions (Lei et al., 2020). Cytotoxic CD8 T cells are the predominant cellular anticancer immune killer cells (Farhood et al., 2019). The important node in immune checkpoint therapy in a mouse model of high TMB breast cancer is the activation of B cells by follicular T helper cells (Tfhs) that mediate the antitumor immune response (Ahmadzadeh et al., 2009). Reactivation of the cellular activity of NK cells, a classical antitumor cell that is evaded by tumor cells, is an extremely promising immunotherapeutic approach (Shimasaki et al., 2020). Our results suggested a correlation between MXD3 and the relative numbers of CD8 T, Tfh, and NK cells, suggesting that MXD3 expression may contribute to the patient’s antitumor response. CD4 T cells can present signals to CD8 toxic lymphocytes and thus mediate antitumor immunity (Zhang et al., 2009). Increasing the number or activating the function of CD4 T cells is also an important direction of immunotherapy (Borst et al., 2018). Cross-presentation of antigen by DCs is thought to be the most potent activation pathway for CD8 cells to activate their cytotoxic killing effect on tumor cells (Wculek et al., 2020). In contrast, in our findings, MXD3 was significantly negatively associated with the number of DCs in almost all cancers. These results suggest that MXD3 may affect the two previously described pathways that activate CD8 T cells and reduce the anticancer immune response. However, this contradicts our previous findings that CD8 T cells were elevated with MXD3, suggesting an intricate relationship between MXD3 and the anticancer immune response activated by immune cells.

Furthermore, our study also revealed the coexpression of MXD3 with genes encoding MHC, immune activation, and immunosuppressive pathways. These results indicate that the expression of MXD3 is closely related to the immune infiltration of tumor cells, affects patient prognosis, and proposes new targets for the development of immunosuppressants.

Hypoplasia is an important pathway in the development of cancer and significantly affects cancer progression, metastasis, and drug resistance (Wong, 2011). In almost all cancers, MXD3 was significantly associated with a variety of apoptosis genes, demonstrating MXD3’s considerable impact on apoptosis. This finding verified that MXD3 inhibits apoptosis, as previously reported in the literature. Due to its remarkable iron dependence, iron-catalyzed necrosis is called ferroptosis in cancer cells. Ferroptosis inducers have been approved by the FDA for cancer treatment (Hassannia et al., 2019). MXD3 had a significant negative correlation with ferroptosis-related genes except for GSS and GPX4 in a variety of cancers, suggesting that MXD3 might have a negative correlation with iron dependence in cancer cells and might be a potential therapeutic target for ferroptosis inducers or a valid indicator of ferroptosis.

Furthermore, the enrichment analyses in 33 types of cancer indicated that MXD3 can potentially impact the regulation of autophagy, the cytosolic DNA sensing pathway, the RIG-I-like receptor, the Toll-like receptor signaling pathway, and antigen processing and presentation. Autophagy can affect cancer in different ways (Levy et al., 2017). As a type of RNA-sensing pattern recognition receptor (PRR) that can stimulate innate immunity (Rehwinkel and Gack, 2020), the role of RIG-I-like receptors in tumor therapy has been confirmed in liver cancer and other cancers (Hou et al., 2014). The cytosolic DNA sensing pathway plays a role in activating immune construction, so it is inferred that this pathway mainly plays a role in tumor inhibition (Kwon and Bakhoum, 2020). However, recent evidence suggests that the signaling pathway may also promote tumor progression and metastasis (An et al., 2019). The antigen presentation pathway can activate NK and CD8^+^ T cells, which play an important role in anticancer immunity (Sánchez-Paulete et al., 2017). These findings also verify the previous results of immune cell infiltration. These pathways further illustrate the role of MXD3 in carcinogenesis and tumor immunity.

The data from the CGGA on MXD3 in gliomas illustrate that MXD3 expression differs significantly in different grades of gliomas and glioma histologic grade. Briefly, the more malignant the glioma is, the higher the MXD3 expression is. Notably, MXD3 expression was significantly lower in patients with 1p/19q and IDH mutations, as well as in wild-type patients with comutations of both. Both are indicators of a good prognosis. Thus, high MXD3 expression indicated a poor prognosis. This is consistent with the previous expression data and the results of the subsequent survival analysis. The methylation status of the MXD3 promoter in patients with different pathological degrees was significantly different. In general, the higher the pathological grade and WHO classification of patients, the lower the methylation level of the MXD3 promoter. The lower the promoter methylation, the worse the prognosis.

Finally, we verified the high expression of MXD3 in gliomas by immunohistochemistry. The expression of MXD3 protein in gliomas was significantly higher than that in normal samples.

In brief, our first pancancer study of MXD3 confirmed the high expression of MXD3 in cancer tissues by TCGA and Oncomine. The function of MXD3 and MXD3 methylation on cancer prognosis was validated: most patients with high MXD3 gene body hypermethylation had better survival, while MXD3 may play opposite roles in different tumors. We discovered a negative relationship between MXD3 and TMB and MSI. We further investigated the relationship between MXD3 and immune infiltrating cells and identified the correlation between MXD3 and immune genes, immunosuppressive genes, and antigen-presenting genes. Finally, we also verified the expression of MXD3 in LGG using IHC. These results suggest that MXD3 is a poor prognostic factor in many cancers, especially gliomas. Although more clinical evidence is needed for MXD3 to be considered a clinical therapeutic target and immunotherapy site, this gene can play an important guiding role in a variety of clinical treatments, including immunotherapy and demethylation therapy.

## Data Availability

The original contributions presented in the study are included in the article/Supplementary Material, and further inquiries can be directed to the corresponding author.

## Supplementary Material

Supplementary Figure S1 Difference in MXD3 expression in multiple tumors between older (>=65) and young (<65) patients.

Supplementary Figure S2 Differential expression of MXD3 in multiple tumors in different sex groups.

Supplementary Figure S3 Expression of MXD3 in different tumor stages.

Supplementary Figure S4 Stromal score (top) and immune score (bottom) in multiple cancer types.

Supplementary Figure S5 GO analysis of the remaining 25 types of cancer by GSEA.

Supplementary Figure S6 Survival curve of the bladder cancer (a, b) and melanoma (c, d) in the ICI cohort.

Supplementary Table S1 Log2 (fold change) of MXD3 between the drug response group and the non-response group across multiple drugs.

Supplementary Table S2 Tumor MXD3 prognosis data obtained from the PrognoScan web tool.
