# Corrigendum: MXD3 as an Immunological and Prognostic Factor From Pancancer Analysis

**DOI:** 10.3389/fmolb.2021.826905

**Published:** 2021-12-22

**Authors:** Xiaoyu Zhang, Xiaoqin He, Yue Li, Yangtao Xu, Wenliang Chen, Xin Liu, Xinyao Hu, Lin Xiong, Ximing Xu

**Affiliations:** ^1^ Cancer Center, Renmin Hospital of Wuhan University, Wuhan, China; ^2^ Department of Pathology, Renmin Hospital of Wuhan University, Wuhan, China

**Keywords:** MXD3, cancer, prognosis, methylation, immune, immune-infiltrating cell

In the published article, incorrect affiliation was included for **Xiaoyu Zhang** ^**1**^ **, Xiaoqin He** ^**1**^ **, Yue Li** ^**1**^ **, Yangtao Xu** ^**1**^ **, Wenliang Chen** ^**1**^ **, Xin Liu** ^**1**^ **, Xinyao Hu** ^**1**^ **, Ximing Xu** ^**1**^. Instead of “^1^Wuhan University, Wuhan, China” it should be ^*1*^ *Cancer Center, Renmin Hospital of Wuhan University, Wuhan, China*. In addition, incorrect affiliation was included for **Lin Xiong** ^**2**^. Instead of “^2^Renmin Hospital of Wuhan University, Wuhan, China” it should be ^2^ *Department of Pathology, Renmin Hospital of Wuhan University, Wuhan, China*.

The authors apologize for these errors and state that this does not change the scientific conclusions of the article in any way. The original article has been updated.

