# Supplementary figures and images for: MXD3 as an Immunological and Prognostic Factor From Pancancer Analysis
Source: Front Mol Biosci. 2021 Nov 11;8:702206. doi: 10.3389/fmolb.2021.702206 (PMC8632067; doi:10.3389/fmolb.2021.702206)

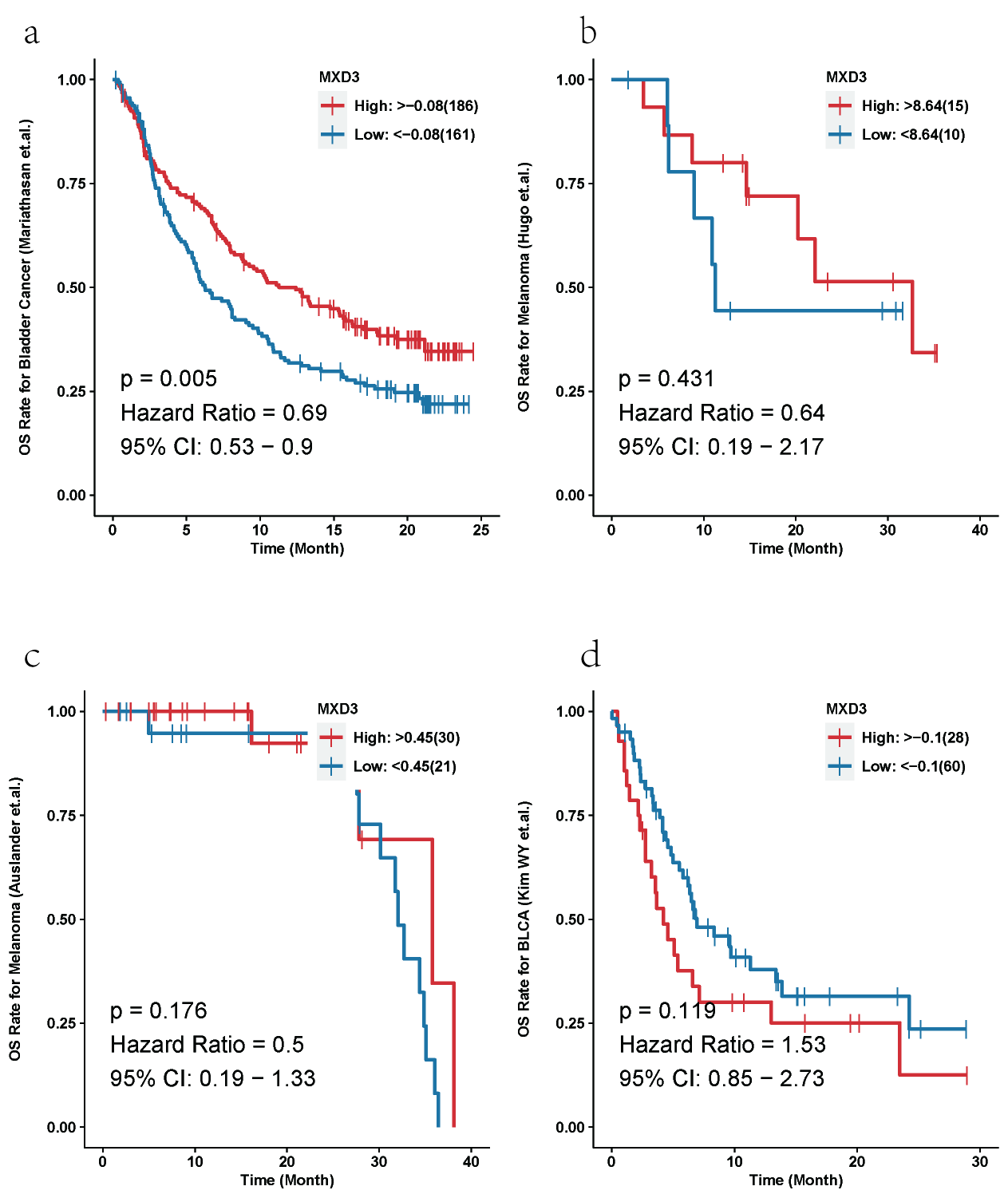

Supplement: Supplementary file 2 [file Image6.TIF]

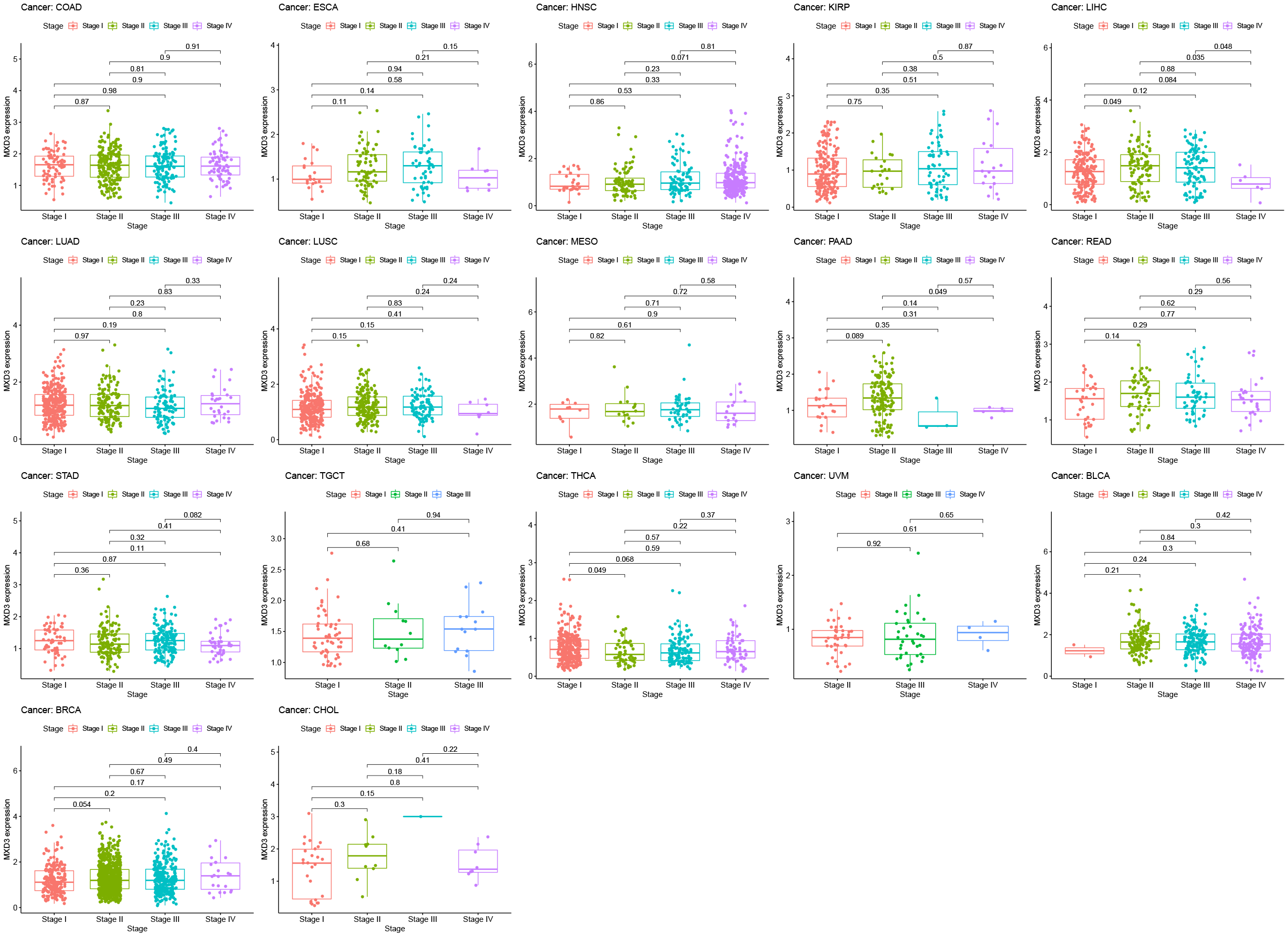

Supplement: Supplementary file 3 [file Image3.TIF]

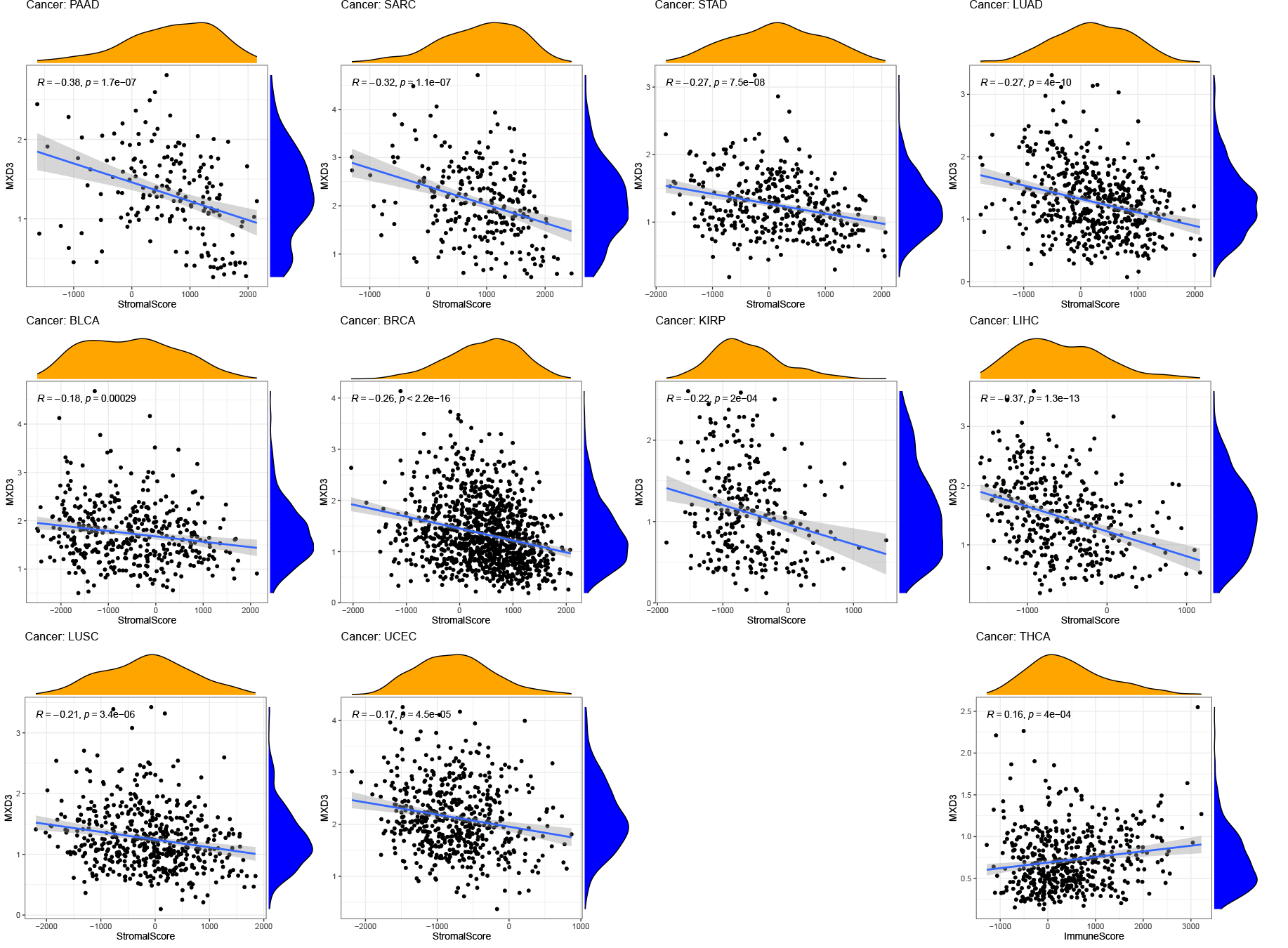

Supplement: Supplementary file 4 [file Image4.TIF]

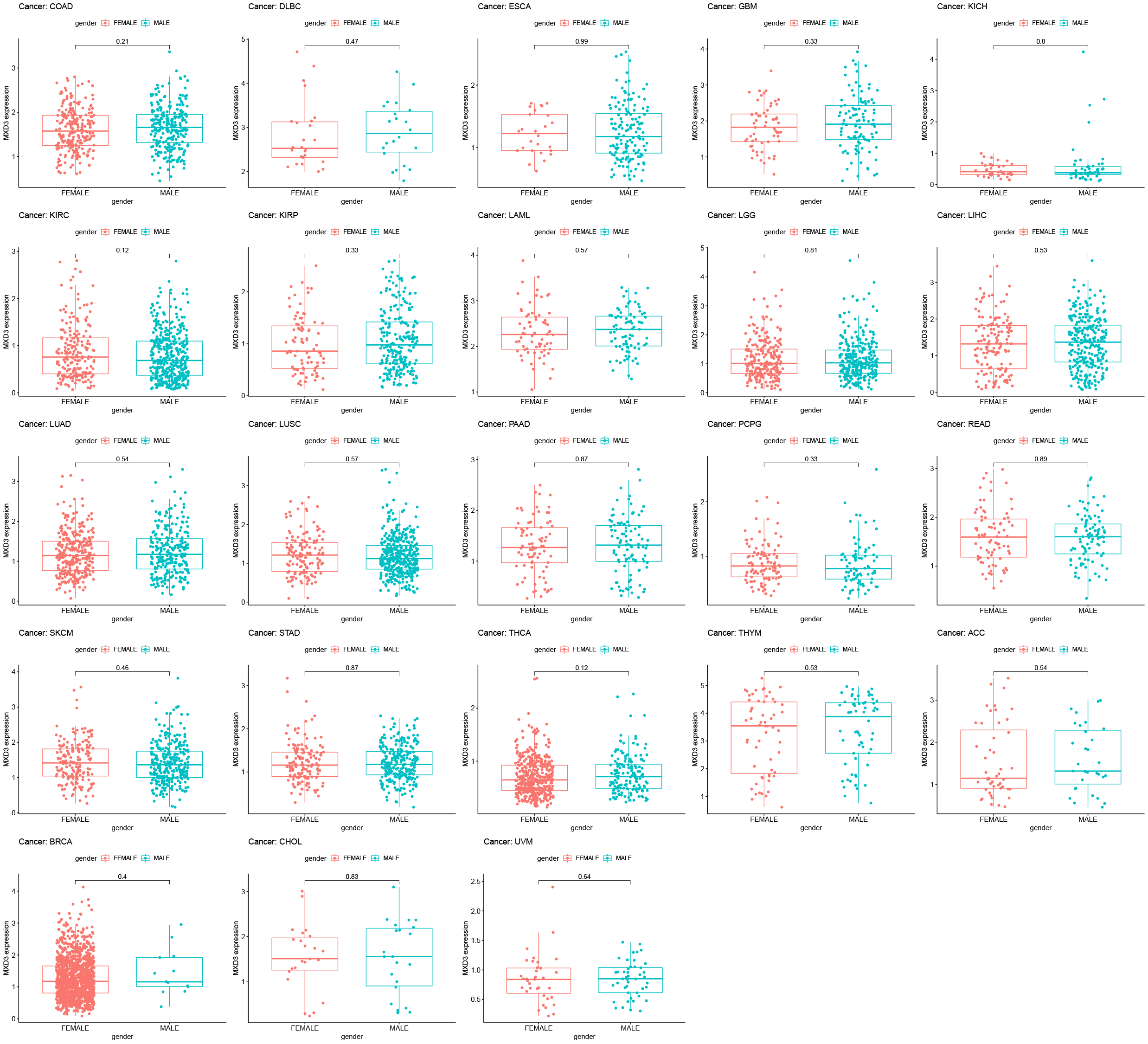

Supplement: Supplementary file 5 [file Image2.TIF]

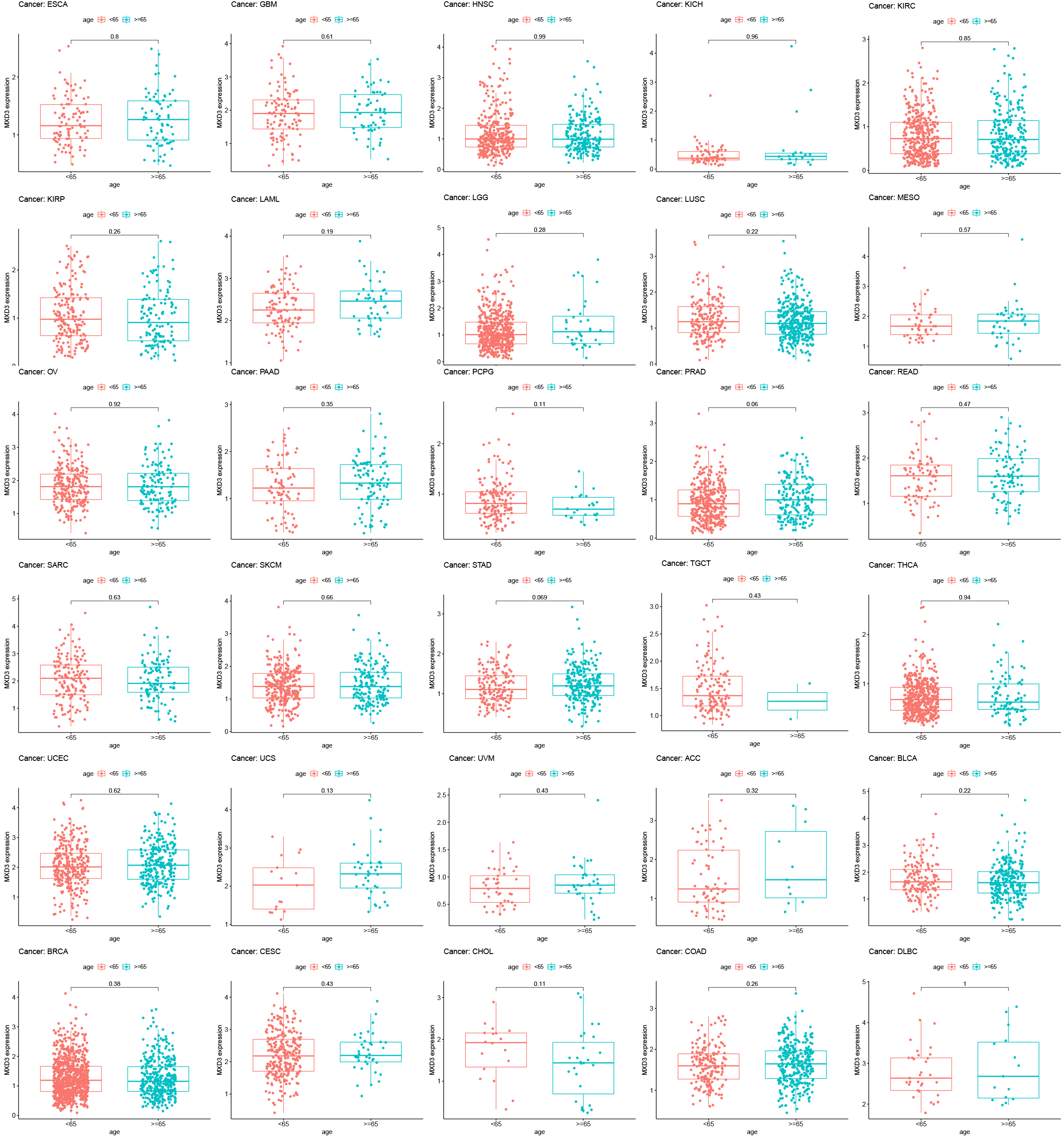

Supplement: Supplementary file 6 [file Image1.TIF]

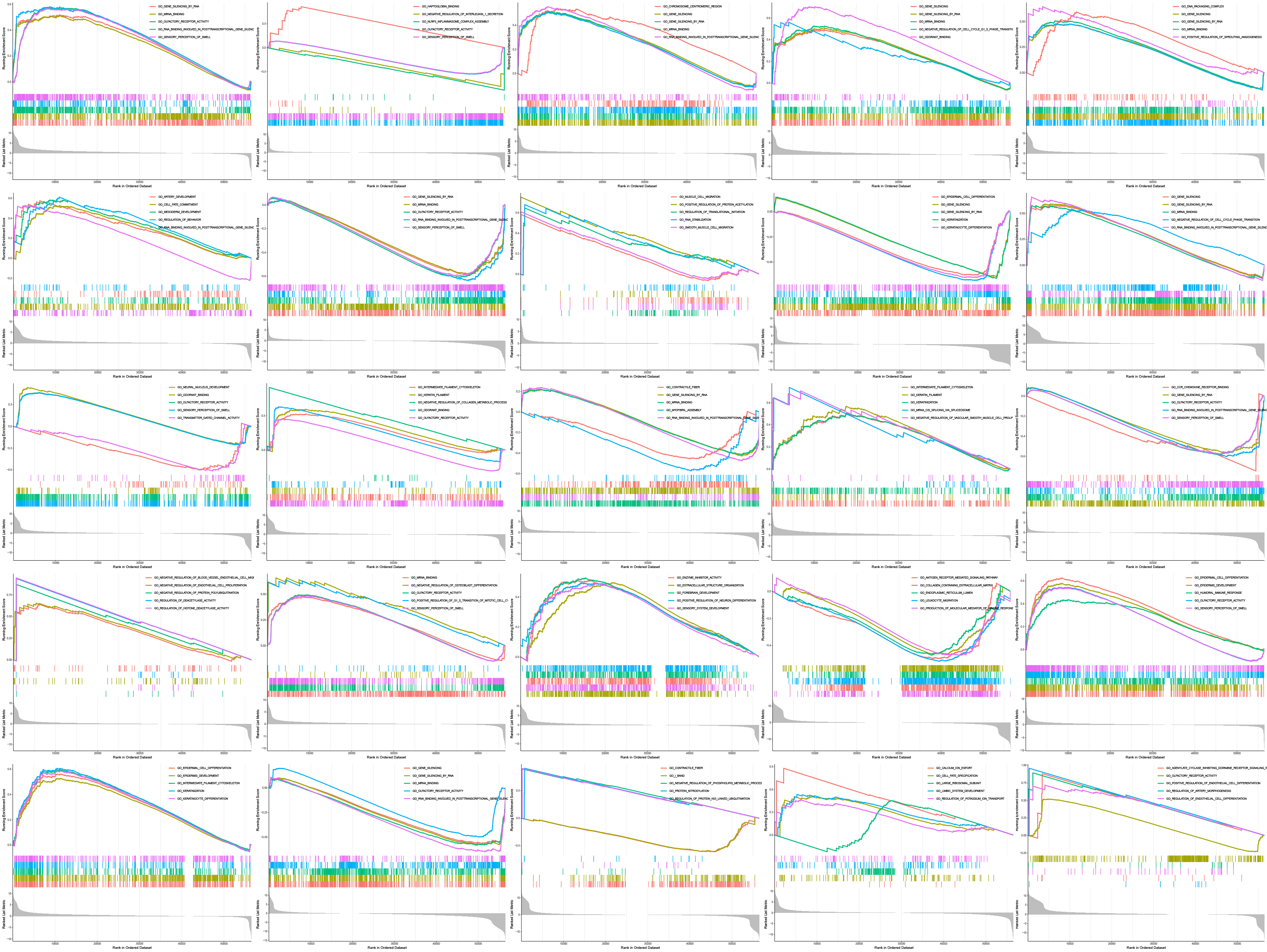

Supplement: Supplementary file 8 [file Image5.TIF]
